# Laser-induced carbonization of cellulose acetate for controlled carbon structure evolution

**DOI:** 10.1039/d6ma00155f

**Published:** 2026-08-03

**Authors:** Angelica Bisceglie, Pietro Zaccagnini, Luisa Baudino, Marco Fontana, Yu Kyoung Ryu, Andrea Lamberti, Javier Martinez

**Affiliations:** a Department of Applied Science and Technologies – Polytechnic of Turin Corso Duca degli Abruzzi 24 Turin 10129 Italy andrea.lamberti@polito.it; b Centre for Sustainable and Future Technologies – Italian Institute of Technology Via Livorno 60 Turin 10144 Italy; c Instituto de Sistemas Optoelectrónicos y Microtecnología, Universidad Politécnica de Madrid Avenida Complutense 30 Madrid 28040 Spain; d Departamento de Física Aplicada e Ingeniería de Materiales, E.T.S.I Industriales, Universidad Politécnica de Madrid C/José Gutiérrez Abascal 2 Madrid 28006 Spain y.ryu@upm.es; e Departamento de Ciencia de Materiales, E.T.S.I Caminos, Canales y Puertos, Universidad Politécnica de Madrid C/Profesor Aranguren s/n 28040 Madrid Spain

## Abstract

Biomass offers a low-cost and sustainable carbon source. Yet, conventional pyrolytic routes remain energy intensive and require harsh processing conditions. Laser-induced carbonization provides a rapid, efficient and green alternative for the conversion of biomass into carbon structures. Building on the growing demand for sustainable alternatives to petroleum-derived polymers in laser writing, we investigate laser-induced carbon (LIC) produced from cellulose acetate (CA) membranes, a widely available biopolymer, *via* CO_2_ laser irradiation. The flame-retardant bis[2-(methacryloyloxy)ethyl] phosphate (BMEP) enables localized carbonization, overcoming CA's poor thermal stability. By tuning laser parameters, namely the defocus distance and the number of passes, we selectively obtain amorphous carbon, activated carbon (AC), graphene oxide (GO), and laser-induced graphene (LIG). Among these, AC fabricated through a double-pass process at a defocus distance of 7.5 mm achieves outstanding electrochemical performance as a microsupercapacitor (μSC) electrode, delivering an areal capacitance of up to 63 mF cm^−2^, an energy density of 3.3 µWh cm^−2^, and a power density of 0.42 mW cm^−2^. These results outperform those of petroleum-based polymer-derived LIG while utilizing a potentially upcycled precursor. This work expands the scope of LIG precursors and offers a versatile platform for engineering sustainable carbon-based electrodes.

## Abstract 2

Laser-induced carbonization of fire-retardant treated cellulose acetate enables controlled carbon materials formation. Resulting activated carbon electrodes outperform cellulose acetate laser-induced graphene in microsupercapacitor application.

## Graphical abstract

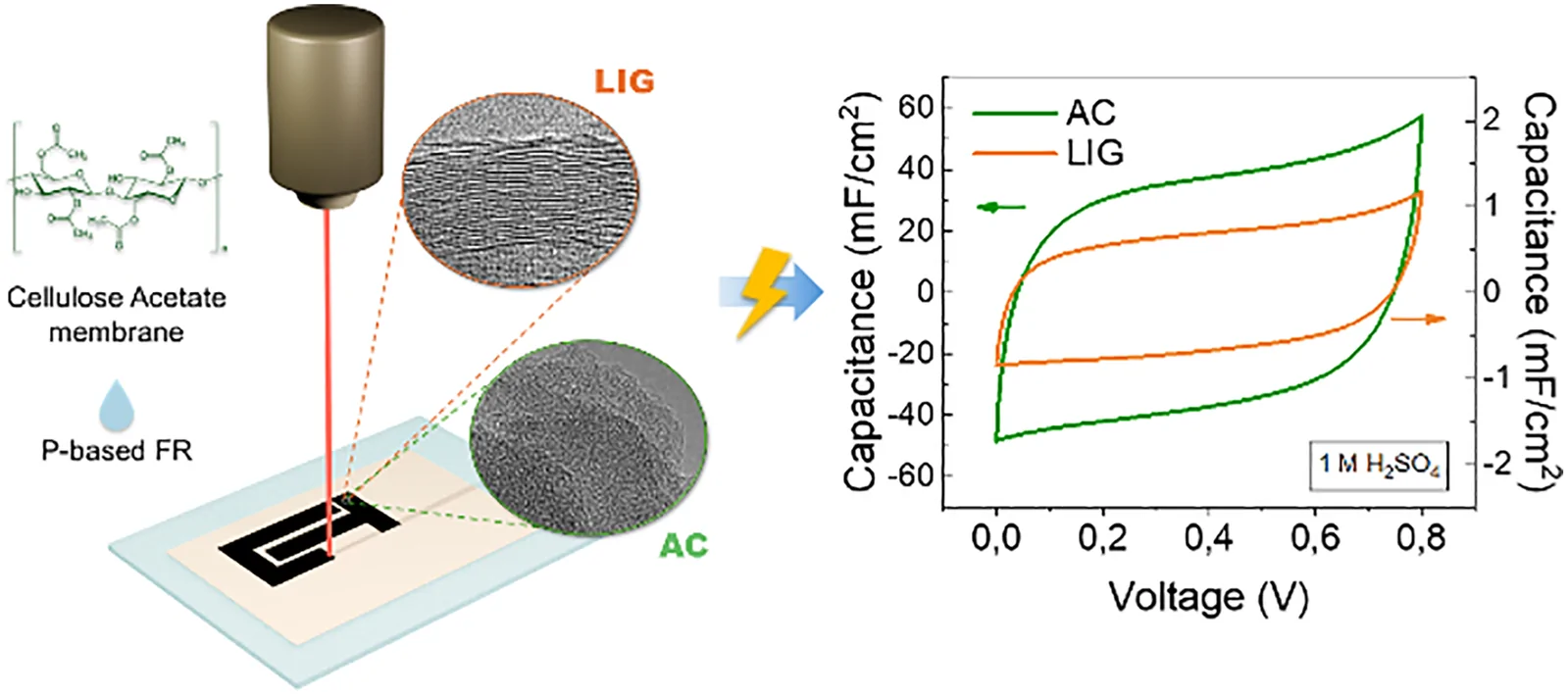

## Introduction

The global transition towards renewable energy sources such as solar and wind has intensified the need for efficient, reliable, and sustainable electrochemical energy technologies capable of mitigating power intermittency. Electrochemical systems for energy storage and conversion, including supercapacitors, batteries, and fuel cells, play a central role in this transition.^1^ Electrode materials are crucial in these applications, since their structure, chemistry, and electronic properties govern device performance, efficiency, and durability. In this context, carbon-based materials have emerged as fundamental components across a broad range of electrochemical applications due to their chemical stability, electrical conductivity, tunable structures, and compatibility with sustainable precursors.^2–4^ Carbon materials exhibit exceptional structural diversity, ranging from highly ordered crystalline graphite to amorphous and highly disordered carbons, with ‘intermediate’ graphene-like materials.^5^ This structural diversity directly translates into different electrochemical behaviors. Graphitic carbons enable ion intercalation and faradaic reactions, making them crucial for lithium- and sodium-ion batteries.^6,7^ Highly disordered and porous carbons predominantly store charge through electrostatic processes, as observed in electric double-layer capacitors (EDLCs).^5,8^ Consequently, tailoring the carbon phase represents a powerful strategy for optimizing electrochemical performance across energy storage and conversion technologies. Among carbon materials employed in EDLCs, activated carbons (ACs) have traditionally been regarded as benchmark electrodes due to their high surface area and microporous structure.^9,10^ However, extensive studies have demonstrated that the specific surface area alone does not correlate with capacitance or energy density. Instead, recent advances have highlighted the critical role of carbon structural disorder in enhancing electrochemical performance.^11,12^ These insights shift the design paradigm from purely morphological considerations toward a more fundamental focus on the carbon phase and the bonding configuration.

Conventional fabrication methods typically involve furnace pyrolysis and activation^9,10,13^ for AC, as well as templated chemical vapor deposition (CVD)^14^ and carbide reactions with halogens^15,16^ for three-dimensional porous graphene-like structures. These processes often require significant thermal energy and prolonged chemical treatments, which can diminish the overall environmental benefits. Current research is therefore increasingly focused on simple, scalable, and environmentally sustainable fabrication strategies for graphene and other carbonaceous materials originating from green substrates.^17^ Particularly, laser-induced-graphene (LIG) has attracted significant attention from the scientific community over the past decade.^18,19^ LIG is a foam-like, 3D porous material composed of disordered graphene sheets that resemble graphene but lack long-range lattice order.^20,21^ It is produced through a rapid, cost-effective bottom-up synthesis process in which a carbon-rich substrate undergoes a laser-induced carbonization (LIC) process through laser beam exposure. The irradiation induces localized temperature ramps of up to 2000 °C, triggering thermal–pyrolytic reactions.^21^ These reactions carbonize the material and release gases, forming the characteristic porous structure of LIG.^18,22^ The versatility of this material has driven its application across a wide range of electrochemical and electronic devices, including sensors,^23–25^ batteries,^26^ supercapacitors,^27,28^ microfluidic systems,^29^ and energy harvesters.^30^ Early studies showed that polyimide substrates could be directly converted into conductive carbon through laser irradiation under ambient conditions, leveraging the aromatic nature of the polymer backbone to promote graphitic domain formation.^31^ Subsequent research expanded the range of suitable precursors, including both synthetic polymers^32,33^ and natural materials.^34^ Biomass-derived substrates such as wood,^35,36^ coconut shells,^37^ and cork^38^ were among the first natural precursors explored due to their high lignin content, which favors aromatic char formation and graphitization under rapid heating ramps. In contrast, polysaccharide-based materials such as cellulose and chitosan generally exhibit lower thermal stability and tend to decompose into volatile species under rapid heating.^39^ To enable effective LIC, chemical pretreatments using phosphorus- or boron-containing compounds have been employed to catalyze dehydration and oxygen elimination, thus inhibiting decomposition into levoglucosan and other volatile compounds, and promoting carbon network formation under ambient conditions.^39,40^ This has allowed the transformation into LIG of paper^23,41,42^ and chitosan.^40,43^ Multiple irradiation strategies have also been introduced to further promote the reorganization of initially formed char into more conductive carbon structures.^41^

Among cellulose-derived polymers, cellulose acetate (CA) represents a widely used yet largely unexplored precursor for LIC. CA is extensively employed in industrial products such as eyewear frames, cigarette filters, and photographic films, generating large volumes of waste with limited biodegradability and significant environmental impact.^44,45^ From a circular economy perspective, identifying value-added applications for CA waste is therefore of considerable interest. Recent studies have demonstrated the recovery and reuse of CA waste for membrane fabrication,^46,47^ as well as its suitability as a precursor for carbon nanotubes and activated carbons through conventional thermal and chemical routes.^48,49^ However, the potential use of cellulose acetate as a precursor for LIC and phase-tailored carbon materials has not been previously reported.

In this work, we demonstrate the use of cellulose acetate membranes as versatile precursors for LIC, enabling the formation of distinct carbon phases ranging from amorphous and activated carbons to graphene-like structures. Rather than focusing on morphology control alone, this study emphasizes carbon material control as a key design parameter governing electrochemical behavior. By correlating the material structure, disorder, and surface chemistry with the electrochemical performance of symmetric supercapacitors, we show that disordered carbon phases derived from cellulose acetate can outperform conventional laser-induced graphene electrodes. These findings highlight the importance of carbon phase engineering and establish laser-induced carbonization as a powerful and sustainable route for designing high-performance carbon electrodes.

## Experimental

### Laser processing parameters

Commercially available cellulose acetate (CA) membranes (90–100 µm thickness, 47 mm diameter, 0.45 µm pore size) by GVS North America (Sandford) were soaked for 15–20 seconds in bis[2-(methacryloyloxy) ethyl] phosphate (BMEP) fire retardant by Sigma-Aldrich (USA) and then dried with a N_2_ blow gun. This allowed for a fire-retardant uptake of 148 ± 20 mg, corresponding to an ≈66 wt% fire retardant content (untreated membrane mass: 77 ± 1 mg). Membranes were then positioned onto glass slides and processed using a 10.6 µm CO_2_ laser (SIHAO, China) with a maximum power of 40 W. Samples were produced varying the lasing parameters, namely the lasing power, scanning speed, distance from the focal plane, and number of lasing passes. The lasing power ranged from 4% to 6% in increments of 0.5%, while the scanning speed varied between 10 and 100 mm s^−1^ in 10 mm s^−1^ increments. Defocus was investigated by varying the distance from the focal plane (−7.5 mm, −4 mm, −3 mm, and 0 mm). Effects of single and double passes were explored. Further details on laser parameters are reported in Table S1.

### Physical–chemical characterization

Optical microscopy images of cross-sections were taken employing a KERN optics OBN 135. Secondary electron scanning electron microscopy (SEM) images were acquired using a FEI Verios 460 (FEI) with an acceleration voltage of 2 kV.

Raman spectroscopy was performed using a LabRAM HR Evolution (Horiba, Japan) employing a 532 nm laser. The focusing of the laser beam was obtained through a Leica 10× objective. Spectra were acquired at an ND filter of 50%, 5 accumulations and 20 s of acquisition time across the range [1100–3000] cm^−1^. Spectrum analysis and baseline correction were performed using the Fityk software.^50^

Peak fitting was performed by modeling the D, G and D′ peaks – where the D′ peaks were distinctly identified using three pseudo-Voigt functions, while Gaussian functions were applied to the D* and D″ peaks, following the most used approach reported in the literature.^51–54^ For the second-order region [2400–3000] cm^−1^, three Lorentzian functions were employed in accordance with prior studies.^52,55^

X-ray photoelectron spectroscopy (XPS) was performed using a PHI VersaProbe II Hybrid system (Physical Electronics, Inc. (PHI), Chanhassen, MN, USA). Monochromatic Al Kα (1486.6 eV) was used as the X-ray source. Different pass energy and step values were used for the survey (187 eV and 0.8 eV) and high-resolution (HR) spectra (23.5 eV and 0.1 eV) acquisition. Charge compensation during the measurements was accomplished with a combined electron and Ar^+^ neutralizer system. Wide-energy and high-resolution (HR) XPS spectra were collected and processed using CasaXPS software (version 2.3.25).^56^ HR spectra were deconvoluted into individual mixed Gaussian–Lorentzian peaks (GL (30)) and asymmetric peaks (LA (1.2,2.5,5)) after Shirley background subtraction and binding energy (BE) calibration. The asymmetric C–C sp^2^ peak in graphitic structures (284.5 eV) was used as the reference for the calibration.

TEM images were obtained using a Tecnai G2 F20 S-twin instrument (FEI) and image elaboration was performed using Digital Micrograph by Gatan. Regarding sample preparation, a dispersion of the lased CA material in high-purity ethanol (>99.8%) was obtained by sonication and subsequently deposited onto a lacey-carbon Cu TEM grid by drop-casting.

### Device assembly and electrochemical characterization

Interdigitated lased-CA electrodes were fabricated under different defocusing conditions, specifically at −3 mm and −7.5 mm. Electrodes were produced employing the double pass technique, setting a laser power of 1.8 W, and scan speeds of 10 mm s^−1^ for the first pass, and 30 mm s^−1^ for the second lasing pass. Various geometries (Fig. S1) were used to study the resulting materials and their corresponding electrochemical performance in 0.5 M H_2_SO_4_. Titanium grids were employed as current collectors while a glass microfiber filter (*t* = 675 µm, Whatman) was used to retain the aqueous electrolyte. A diagram of the assembly is presented in Fig. S2.

Electrochemical characterization was performed in a two-electrode configuration using a multichannel potentiostat (VMP3, BioLogic). The measurements included electrochemical impedance spectroscopy (EIS), performed in the frequency range of 10 mHz to 1 MHz with 10 points per decade and a voltage amplitude of 5 mV; cyclic voltammetry (CV), conducted at scan rates of 10, 20, 50, 100, and 200 mV s^−1^; and galvanostatic charge–discharge (GCD) measurements, carried out at current densities of 0.1, 0.2, 0.5, 1, and 2 mA cm^−2^. Capacitance values were determined from GCD tests by evaluating the discharge capacity (*Q*_d_) and discharge energy (*E*_d_) of the system using eqn (1):1
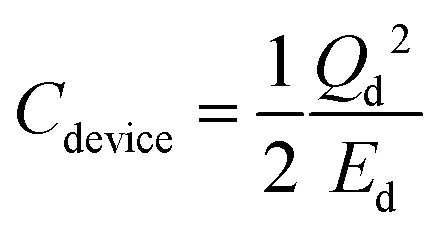


To account for the effects associated with the selected electrode geometry and ensure comparability of the results, the specific capacitance normalized to the electrode area exposed to the electrolyte, *S*, was also calculated as shown in eqn (2):2
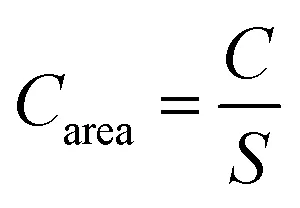


The electrode area *S* exposed to the electrolyte taken into consideration was obtained from the laser path files. The specific energy and specific power were determined from the GCD curves. The first was calculated by dividing the energy provided by EC-Lab software by the exposed area, as expressed in eqn (3):3
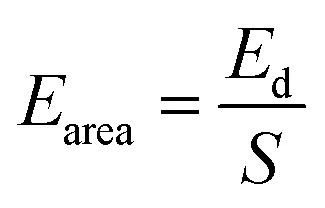


Instead, the specific power was evaluated from the energy and the time of discharge as shown in eqn (4):4
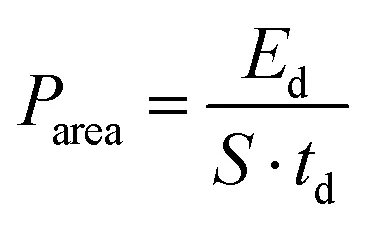


## Results and discussion

### Physical–chemical characterization

Preliminary studies on the effect of the BMEP fire retardant and lasing parameters were performed on 3 × 3 mm^2^ areas of the CA membranes irradiated with a CO_2_ laser scanned continuously along parallel lines. As a first test, untreated membranes were subjected to the lasing process, resulting in the complete ablation of the material. When exposed to CO_2_ laser radiation, the material surface reaches local temperatures up to 2000 °C, with consequent degradation and volatilization of the untreated CA (Fig. S3). For this reason, BMEP was employed as a fire retardant, inhibiting the decomposition of the cellulosic membrane in such rapid heating conditions thanks to the presence of a phosphate group at the center of its monomers. As previously discussed in the literature for cellulose, the incorporation of a phosphate- or boron-based compound induces phosphorylation or borylation of cellulose. This modification catalyzes dehydration and oxygen elimination reactions, inhibiting its decomposition into levoglucosan and other volatile compounds upon rapid heating under ambient conditions.^39,41^ Indeed, immersing the membranes into the fire retardant significantly improved their thermal properties under different lasing conditions (Fig. S3b). It was observed that the minimum power required to achieve material transformation in all cases was 1.6 W. The optimal results were achieved using a power setting of 1.8 W at a scanning speed of 10 mm s^−1^, yielding black squares with minimal substrate damage, as highlighted in Fig. S3b with an orange square. Similarly, dosing tests revealed that samples fabricated *via* a double-pass technique, employing 1.8 W of power, 10 mm s^−1^ for the first pass, and 30 mm s^−1^ for the second lasing pass, exhibited superior performance, as evidenced by visual inspection of the specimens (Fig. S3c).

**Fig. 1:**
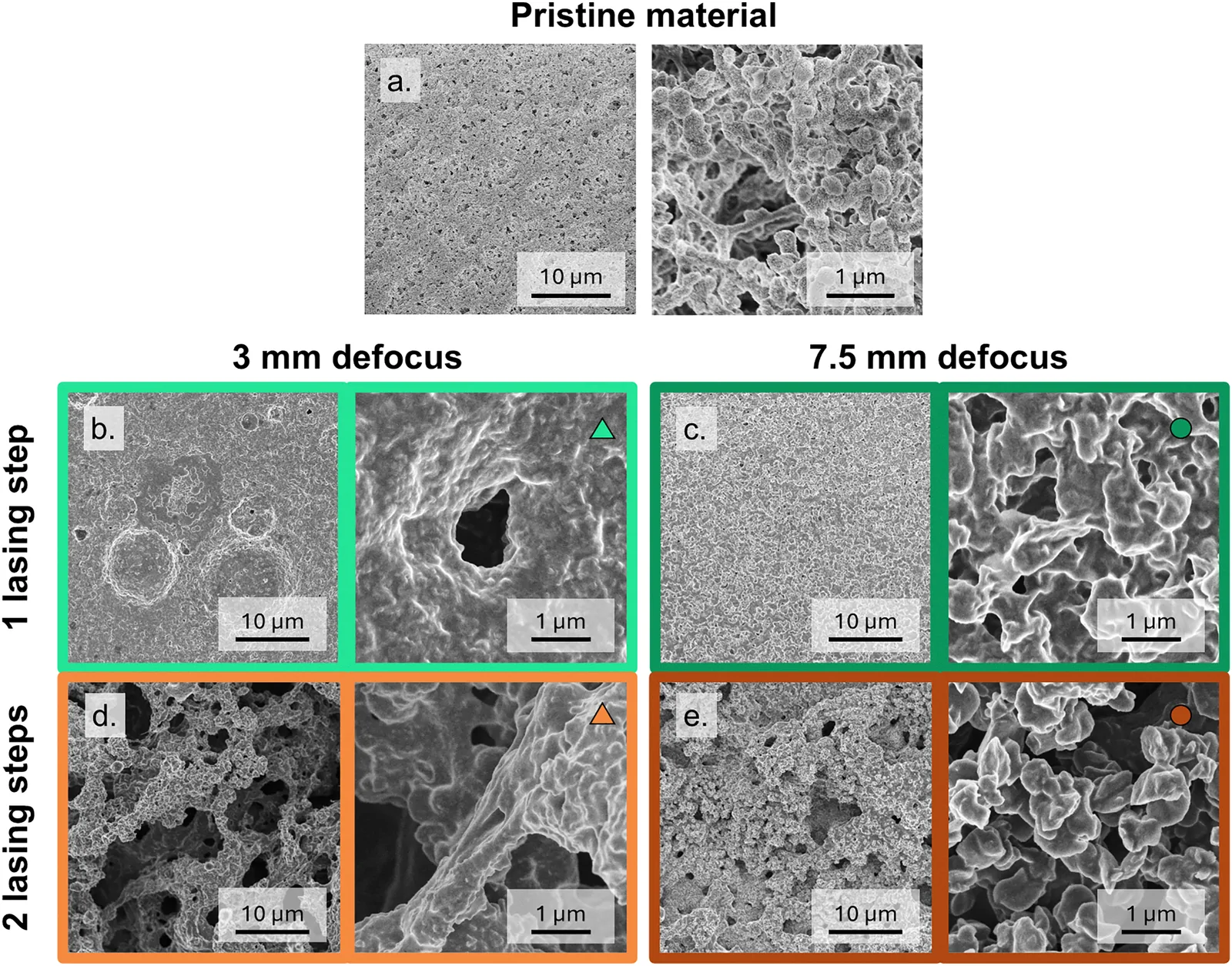
SEM micrographs showing the morphology evolution of CA membranes at two different magnifications. SEM images of the (a) pristine CA membrane; SEM images of samples formed during the first irradiation step at (b) 3 mm and (c) 7.5 mm below the focal plane; SEM micrographs of samples upon the second irradiation step at (d) 3 mm and (e) 7.5 mm.

Fig. 1 shows a morphological comparison between the pristine CA membrane (Fig. 1a), samples produced with a single irradiation step (Fig. 1b and c) and samples produced with two irradiation steps (Fig. 1d and e). Further insights into the impact of the distance from the focal plane are provided in Fig. S5.

The pristine CA membrane (Fig. 1a) exhibits a uniform macroporosity of 1 µm^−2^ µm and a net structure with elements of approximately 200 nm. As illustrated in Fig. 1b and c, after the first laser irradiation, the structure becomes denser. Notably, the sample produced at 3 mm of defocus (Fig. 1b) is characterized by a morphology that appears to be remarkably different from the one observed for the sample irradiated at 7.5 mm of defocus (Fig. 1c). While samples processed closer to the focal plane appear denser (Fig. 1b), those treated farther from the focal plane retains the morphology more similar to that of the untreated membrane (Fig. 1c). This considerable difference in morphology at varying defocus levels could be attributed to changes in fluence experienced by the material when altering the lasing plane. Upon the second, faster lasing pass, additional energy is imparted to the previously formed carbonaceous material. Double lased samples (Fig. 1d and e) exhibit greater porosity than those produced in a single pass (Fig. 1b and c). This increased porosity results from the degassing process that facilitates the release of oxygen-containing gaseous compounds. In Fig. 1d, the sample lased at 3 mm of defocus is characterized by a highly porous structure with thin, interconnected, network-like features which, at higher magnifications, display a rough surface. As illustrated in Fig. 1e, the sample produced at 7.5 mm of defocus displays a randomly and uniformly distributed porosity ranging from 2 to 10 µm, along with a granular structure. Inspection at higher magnifications makes it possible to observe a homogeneous and smooth surface with visible particle agglomerates characterized by rough surfaces. A comparison of these two morphologies makes it evident that a lasing process closer to the focal plane produces a more porous material, likely because of a more powerful degassing process.

**Fig. 2:**
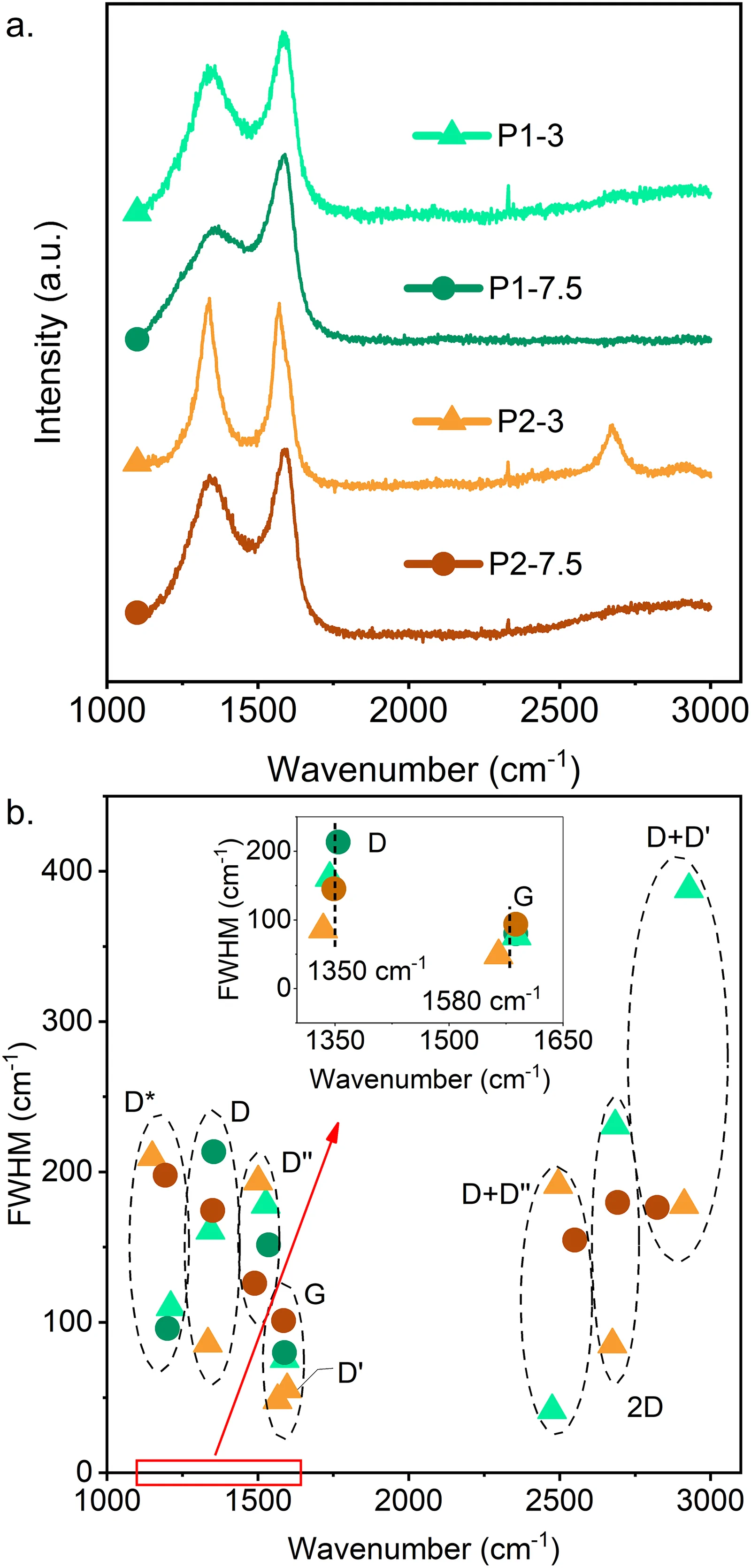
(a) Raman spectra of samples produced in one irradiation step and two irradiation steps at different distances from the focal plane. (b) The correlation between the peak position and FWHM derived from peak deconvolution. Dashed lines represent the theoretical positions of D and G peaks in graphitic materials. The inset provides an enlarged view of the main peaks in the first order region.

Further insights into the transformation process have been extrapolated from Raman spectra analysis, as shown in Fig. 2 and further detailed in Fig. S6, Table S2 and Fig. S7. To improve the visualization of information within the panels, the samples have been labeled as *P*_*x*−*y*_, where *x* indicates the number of passes and *y* represents the distance of the lasing plane from the focal plane in millimeters.

Raman spectra of carbonaceous materials typically feature three main peaks: the G peak (∼1580 cm^−1^), associated with in-plane stretching of sp^2^ carbon atoms (E_2g_ phonon mode);^55,57–59^ the D peak (∼1350 cm^−1^), arising from breathing modes of six-membered rings and activated by structural defects;^55,57,58^ the 2D peak (∼2700 cm^−1^), an overtone of the D peak.^51,55^ While the G and D peaks are consistently observed in all the spectra of Fig. 2a, the 2D peak can present indistinct features or be totally absent depending on the degree of disorder. Additional Raman modes D*, D′, and D″ appear in the 1100–2000 cm^−1^ range^52,55,57^ while D + D″ (∼2500 cm^−1^) and D + D′ (∼2950 cm^−1^) combinations are observed in the second order region.^51,52,55,59,60^ Samples irradiated once (P1-7.5 and P1-3) lack a clear D′ peak near the G peak, placing them in Stage 2 of the amorphization trajectory of carbon materials,^58^ where an increasing amorphization corresponds to a decreasing *I*_D_/*I*_G_ ratio. In particular, P1-7.5 with an *I*_D_/*I*_G_ ratio of 0.81 displays a greater degree of amorphization and can be assimilated to amorphous carbon (a-C).^58^ This appears to be in great accordance with the SEM micrographs (Fig. 1c), as a greater distance from the focal plane causes a lower energy density to be delivered onto the material surface, leading mainly to carbonization and amorphous char formation. The sample P1-3, instead, with a ratio equal to 1.06 falls into the range typical of nanocrystalline graphite (NC–graphite)^58^ and its spectrum also exhibits features that may be recognized in graphene oxide (GO) spectra.^51,52,60^ Similarly, SEM micrographs reveal substantial agglomeration (Fig. 1b), likely induced by inter- and intramolecular forces facilitated by the high content of oxygen- and hydrogen-containing functional groups in GO. Double irradiation samples show higher degrees of structural order (Fig. 2a). P2-7.5 displays an undifferentiated bump in its second order region and lacks a D′ peak, placing it again in Stage 2 of the amorphization trajectory^58^ and suggesting the presence of GO^51,52,60^ or activated carbon (AC).^59,61,62^An increase of the *I*_D_/*I*_G_ ratio after the second irradiation (from 0.81 to 0.97) also indicates the rearrangement of previously formed sp^2^ clusters into aromatic rings without full ordering.^58^ However, the size of these clusters does not appear to change after the second pass, as the FWHM of the G peak remains nearly unchanged (Fig. 2b).^57,58^ P2-3, instead, exhibits a less pronounced shoulder between the D and G peaks, distinct D + D′, 2D, and D + D″ peaks and a clear D′ peak,^57^ suggesting the presence of laser-induced graphene (LIG).^41,63–66^. This is confirmed by an increase of the *I*_D_/*I*_G_ ratio to 1.34 and is consistent with the highly porous morphology observed in Fig. 1d, compared to the dense structure in Fig. 1b. Furthermore, the G peak downshift from the typical 1580 cm^−1^ position (Fig. 2b),^58,67^ along with halved FWHM of D and G peaks after the second lasing pass, suggests the increased sp^2^ cluster size^58^ and a possible residual tensile stress which elongates graphene sheets and softens vibrational modes.^60,68^ This stress may originate from thermal effects induced during laser interaction with the substrate and the glass support.^58^

**Fig. 3:**
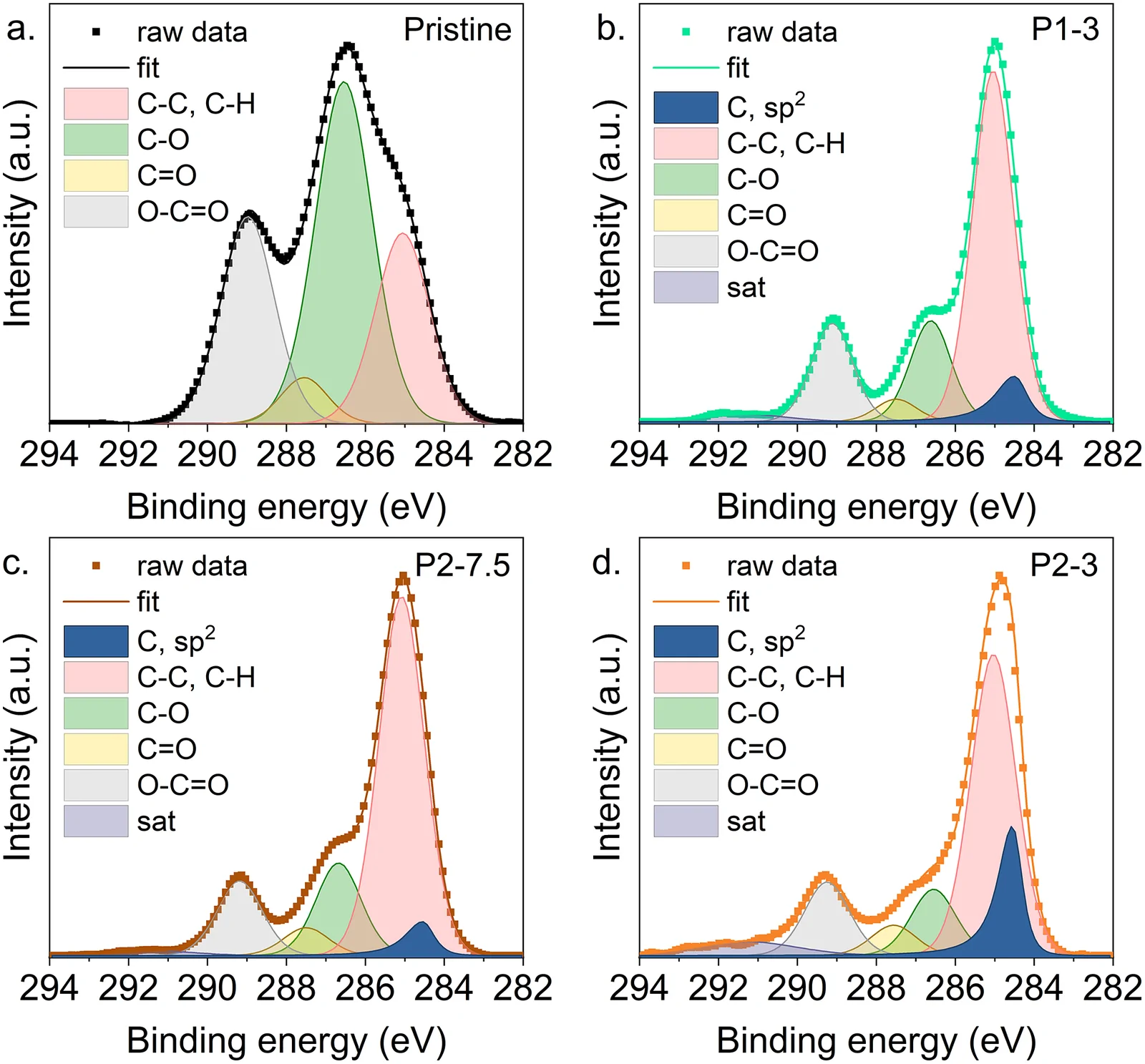
High resolution normalized XPS spectra of the C 1s peak of (a) pristine cellulose samples and samples produced with the (b) one-step irradiation process at 3 mm and two-step irradiation process at (c) 7.5 mm and (d) 3 mm below the focal plane.

XPS survey analyses initially revealed traces of nitrogen, and a non-negligible presence of phosphorous, *i.e.* the fire retardant, still on the surface of all the lased samples (Table S3). The presence of the fire retardant still present on the lased samples could dampen the graphitization signature after laser treatment. Silicon was also found in all samples, with traces in samples lased with only one pass and a few atomic percentages in those subjected to two passes. This had already been documented in the literature in the case of multiple lasing processes on chitosan substrates, and attributed to a partial interaction of the laser beam with the glass substrate.^69^ Coherently with the fact that CA mainly undergoes ablation when subjected to laser writing, the interaction with the glass substrate appears to be more present after the second pass. Furthermore, it appears that after the second pass, the relative amount of carbon significantly decreases. Although a relative decrease was expected due to the partial ablation of CA when subjected to laser writing, this reduction appeared more significant than what the authors would have expected. Normalized high resolution scans of the C 1s region with peak deconvolution of the pristine cellulose acetate membrane and of the most significant lased samples are reported in Fig. 3. Normalized C 1s HR spectra were deconvoluted into the usual components for carbon-based materials with a graphitic component,^70^ with the asymmetric C sp^2^ peak at 284.5 eV as the calibration peak. In the case of the pristine cellulose acetate sample, the position of the peaks was assigned in order to be coherent with the lased samples, hence with the C sp^3^ peak at 285.0 eV. The full details of the position and relative amount of the components are presented in Table S4. As expected from natural polymers, all samples present a considerable fraction of C–O and C=O surface functional groups (respectively, the green peaks at 286.6 eV and the yellow ones at 287.5 eV in Fig. 3) with respect to synthetic polymers.^71–73^ Their amount is significantly reduced after the laser treatment but does not seem to vary much as a function of either the number of passes or the defocus distance. One can also appreciate the presence of a significant amount of carboxyl groups (at 289.1 eV, as depicted in gray in Fig. 3), typical of both cellulose acetate and of the fire retardant, which are not reduced during the lasing processes.^74^

As previously said and evidenced by the presence of phosphorous in the survey spectra, *i.e.* the fire retardant, some of the signals typical of the carbonization processes might be dampened. Indeed, peaks related to aliphatic carbons (at 285.0 eV and rose-colored in Fig. 3) that are minoritarian in cellulosic structures are now predominant in all lased samples. It is however possible to observe some differences in the sample surface composition depending on the number of passes and the defocus distance. By focusing first on the number of passes, it is possible to see that the second pass seems to favor the graphitization process, as evidenced by the increase of the sp^2^ component (depicted in dark blue, at 294.5 eV) in Fig. 3d with respect to Fig. 3b. The relative amount of graphitic carbon indeed doubles, as can also be seen in Table S4, and complementary the amount of C–O bonds is reduced by a third after the second pass.^72^ This is well in accordance with what was found by Raman spectroscopy which indicated the possibility of having a LIG structure at low defocus distances after two passes. This is also evidenced by a more prominent presence of the satellite peak present at 291.3 eV (in violet in Fig. 3). Instead, at higher defocus distances, one appears to have a less graphitic structure even after two passes, with only around 6% of graphitic carbon, a value lower than the one obtained at smaller defocus distances after only one pass, and significantly more oxygen bonds, which should allow for a more hydrophilic surface.

**Fig. 4:**
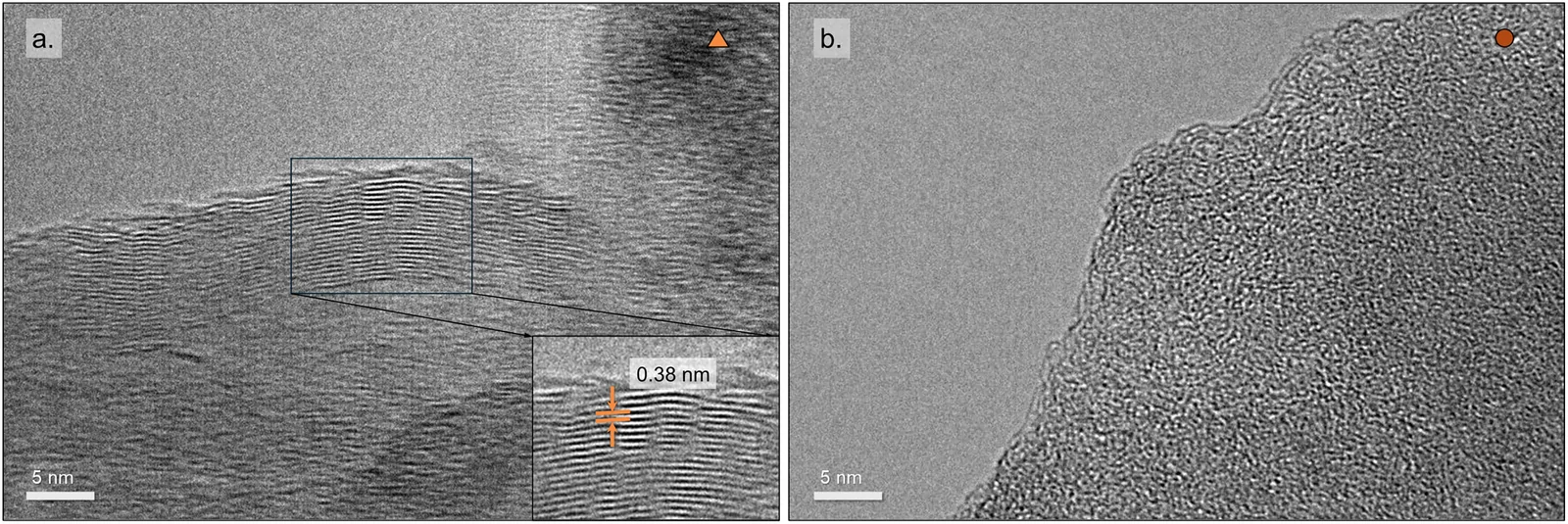
HR-TEM images of samples produced with the two-step irradiation process at (a) 3 mm and (b) 7.5 mm below the focal plane.

These hypotheses were confirmed *via* TEM analyses. The micrographs revealed the presence of domains with a multilayered lamellar structure in the sample produced 3 mm below the focal plane (Fig. 4a), supporting the hypothesis that the second lasing pass leads to the formation of a graphite-like material. The average interplanar distance found was *d*_avg_ = 0.38 ± 0.02 nm, in line with other results in the literature.^73,75^

Comparing the morphological analysis conducted through SEM with Raman spectroscopy results allows for a description of the material's evolution, which is further clarified by the characterization provided by HR-TEM. When the material is closer to the focal plane, it undergoes a more effective transformation: the graphene oxide (GO) formed during the first pass, characterized by few open pores and protrusions likely resulting from closed porosity, is subsequently reduced to LIG in the second pass, with gasification producing elevated porosity and ordering of the material into domains of multiple graphene layers. Conversely, at greater defocus distances, amorphous carbon is formed, characterized by a morphology that results in being very similar to the pristine membrane. Following the double pass, the sample displays a more ordered structure likely due to the formation of activated carbon upon the second radiation exposure. This can be confirmed by the high disorder also observed in the TEM image. In contrast, at 7.5 mm of defocus, the structure is highly disordered (Fig. 4b), where no well-defined graphene layer was found, typical of an amorphous phase. Therefore, rather than GO, this material appears to be activated carbon, likely formed due to the high temperatures reached at the substrate surface induced by the CO_2_ laser combined with the presence of phosphorus from the fire retardant.

In contrast, at 7.5 mm of defocus, the structure is highly disordered (Fig. 4b), where no well-defined graphene layer was found, typical of an amorphous phase. Therefore, rather than GO, this material appears to be activated carbon, likely formed due to the high temperatures reached at the substrate surface induced by the CO_2_ laser combined with the presence of phosphorus from the fire retardant.

### Electrochemical characterization

**Fig. 5:**
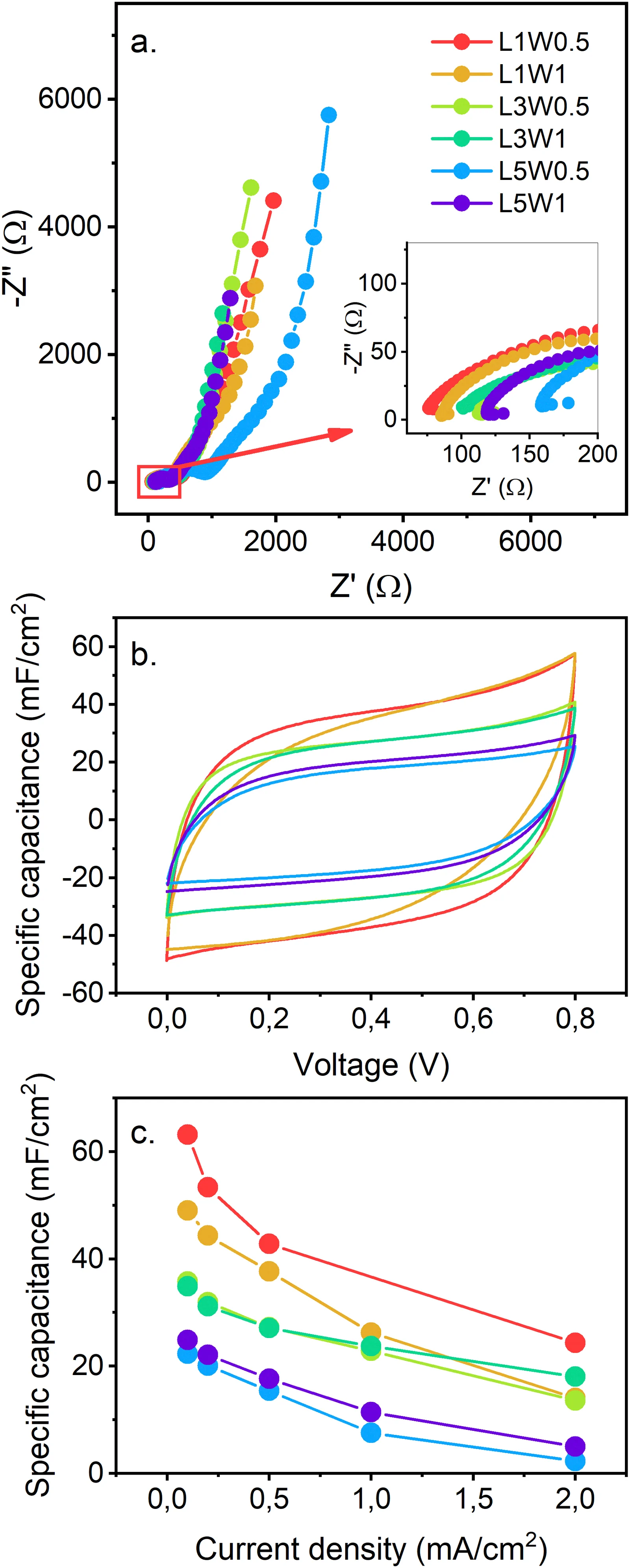
Electrochemical characterization of devices using P2-7.5 as the electrode material. (a) Nyquist plot, (b) CV curve recorded at 10 mV s^−1^, and (c) specific capacitances from GCD curves.

Following material characterization, symmetric supercapacitor devices were fabricated using interdigitated electrodes. Notably, samples synthesized under P2-7.5 lasing conditions exhibited superior electrochemical performance compared to those fabricated under P2-3 conditions. For this reason, the study concentrated on the P2-7.5 parameter set, which led to the formation of AC. To systematically assess the influence of electrode geometry on device performance, the dimensions of the electrodes’ fingers were varied. Specifically, the finger lengths (*L*) ranged from 1 mm to 5 mm in increments of 2 mm, while the width (*W*) was either 1 mm or 0.5 mm, as detailed in Fig. S1a. In the following, samples are therefore designated as *L*_*x*_*W*_*y*_, where *x* denotes the finger length and *y* denotes the finger width. The device assembly procedure is detailed in Fig. S2. Electrochemical testing was performed in a two-electrode configuration using a 0.5 M H_2_SO_4_ aqueous electrolyte and the corresponding results are presented in Fig. 5. For comparison, electrodes fabricated under P2-3 lasing conditions (Fig. S1b) were assembled using the same methodology as the AC ones and subjected to identical electrochemical testing. The electrochemical characterization of LIG devices is reported in Fig. S8. EIS results are presented in Fig. 5a, with further analysis provided in Fig. S9, Tables S5 and S6 and Fig. S10. An electrochemical capacitor can be modeled as an ideal capacitor in series with an equivalent series resistance (ESR), which primarily arises from the combined contributions of electrolyte resistance, interparticle resistance within the active material, and the contact resistance at the interface between the active material and the current collector.^76,77^

As presented in Fig. 5a and as reported in Tables S5 and S6 and Fig. S10, the ESR generally decreases with decreasing finger length in AC interdigitated electrodes, compatibly with Ohm's law prediction. At high frequencies, it is possible to observe a wide semicircle for all samples, which may be attributed to limited electronic conductivity between the carbon particles.^76,77^ In the low-frequency range, a consistent vertical increase in the imaginary part is observed, indicating a capacitive system (reflective boundary^77^). A diffuse region at intermediate frequencies is observed for all systems, which can be ascribed to the restricted motion of ions within the porous structure of the electrodes.^76^ Thus, the primary observation from electrochemical impedance spectra is that the material shows capacitive behavior with a high resistive contribution. Notably, comparative analysis of the Nyquist plots for AC electrode devices (Fig. 5a) and LIG electrode devices (Fig. S8a) demonstrates that the diffusive region amplitude for LIG-based devices is almost equal to the total real component amplitude of the AC ones. This suggests higher energy losses in LIG electrodes arising from reduced ionic transport efficiency within their porous architecture. The areal capacitances derived from the EIS analyses and reported in Table S5 underestimate the actual areal capacitances rated during both voltammetric and galvanostatic experiments. The explanation for this incongruence arises from considering that the reported EIS measurements were recorded under open voltage conditions. Therefore, contributions arising from pseudocapacitive effects due to surface functional groups might not be accounted for. As reported in the XPS analyses, both quinone (C=O) and carbonyl (O–C=O) groups are present on the surface of the AC samples usually considered for their potential faradaic contribution throughout the whole voltage window.^78^

Values obtained from the normalization of CV curves with respect to the scanning voltage are presented in Fig. 5b. This representation enables the plotting of areal capacitance as a function of voltage, revealing that the capacitance decreases with increasing finger length, while the width has a negligible effect. This trend in capacitance retention is likely due to the resistive properties of the material. Beyond a certain length, significant electrode resistance limits its functionality, which may be attributed to the restricted conductivity of the LIC.

Additional representations of CV measurements provided in Fig. S11 offer further insights into electrochemical behavior. Fig. S11a illustrates CV cycles at the lowest scan rate (10 mV s^−1^), which exhibit a predominantly rectangular shape. However, deviations from the ideal rectangular profile are observed due to the resistive contributions, consistent with earlier EIS results. Fig. S11b illustrates current density *versus* voltage curves, indicating that the finger length significantly influences current density during electrochemical cycling within the selected voltage window. Except for L1W1, all samples demonstrate rapid responses at the limits of the voltage operational window despite characteristic resistive behavior across all cells. Fig. S11c further confirms this trend by normalizing the current axis to peak values. Galvanostatic charge–discharge measurements in H_2_SO_4_ (Fig. 5c) show a clear decrease in areal capacitance with increasing electrode length, further supporting the trends previously observed in the areal capacitance values derived from cyclic voltammetry curves (Fig. 5b). The capacitance behavior at low current densities is also consistent with the observations from electrochemical impedance spectroscopy results (Fig. 5a and Fig. S10), where lower ESR values correspond to higher capacitive behavior. The comparison with LIG electrodes reveals that AC electrodes are characterized by a capacitance that is one order of magnitude higher (Fig. 5c and Fig. S8). This can be explained by the findings of Liu *et al.*,^11^ who demonstrated that capacitance is enhanced in carbon systems with greater disorder.

**Fig. 6:**
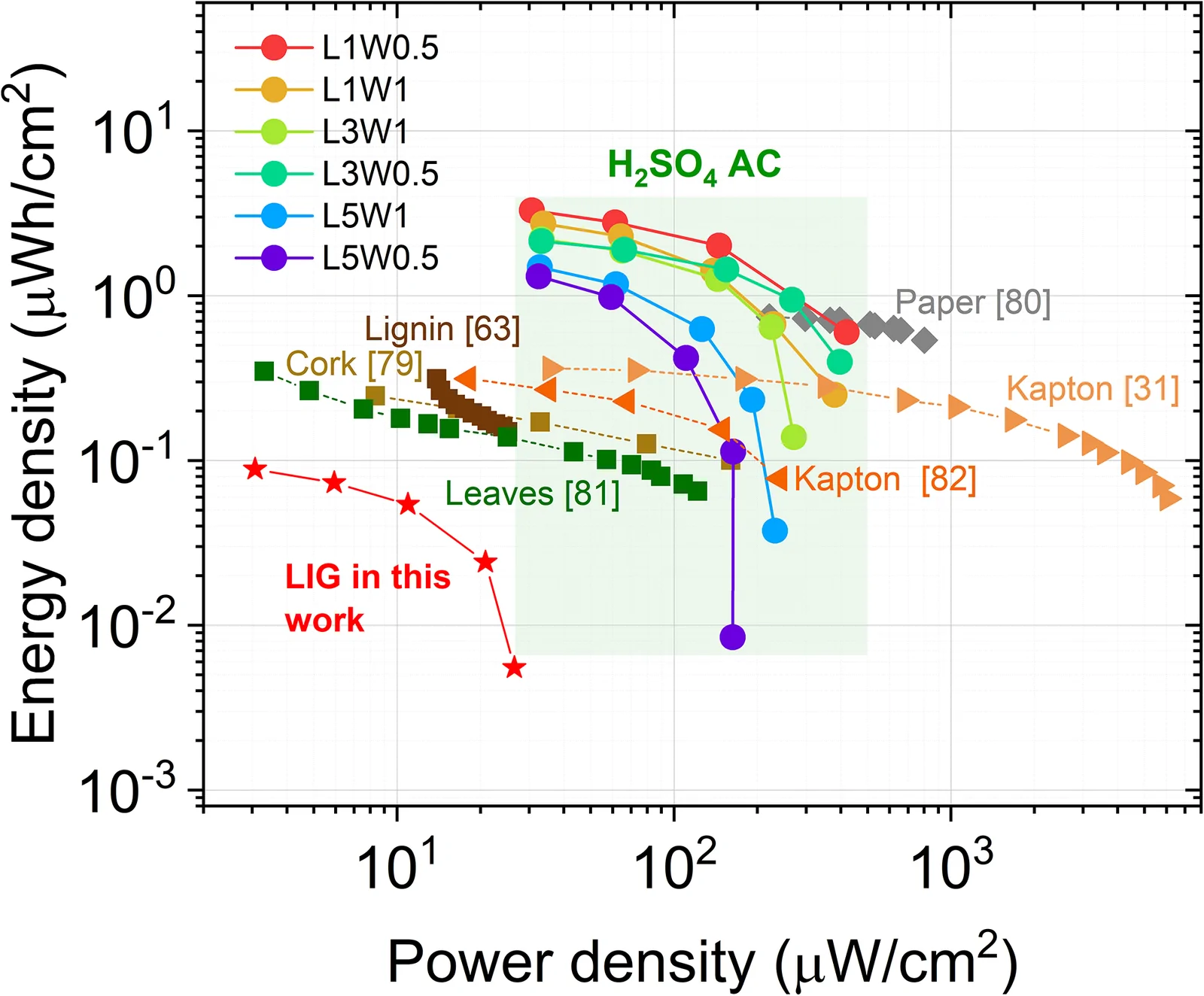
Ragone plot comparing the performance of the devices of this paper with bio-derived^63,79–81^ and Kapton-based^31,82^ LIG supercapacitors from the literature.

Fig. 6 presents the Ragone plots for all the devices tested in H_2_SO_4_ (0.5 M), with power and energy densities evaluated from the GCD measurements. The highest performing device is the one produced under P2-7.5 conditions with *L* = 1 mm and *W* = 0.5 mm, as expected. This behavior can be explained by the direct proportionality between energy density and the system's capacitance, while power is inversely proportional to the system's equivalent series resistance (ESR). Further analysis of the Ragone plot reveals that the investigated electrodes demonstrate higher performance under low power densities when compared to state-of-the-art devices^63,79–81^ with LIG electrodes from green materials, achieving energy densities nearly one order of magnitude greater. The best performing device featured an energy density of 3.3 µWh cm^−2^ at low power rates and a maximum power density of 0.42 mW cm^−2^. Furthermore, the devices developed in this work outperform LIG devices fabricated from Kapton,^31^ as well as supercapacitors employing LIG from Kapton enhanced with activated carbon,^82^ in terms of energy density. Based on the Ragone plot in Fig. 6, it can be inferred that these devices are functional towards integration in harvesting systems and direct system powering in the absence of primary energy sources.^83^ Finally, this work presents an attractive process to synthesize activated carbon electrodes with the desired geometry directly from the precursor through laser writing in addition to the most extended method of carbonization and chemical activation as powder.^84,85^

## Conclusions

In this study, different carbon-based materials were successfully synthesized by irradiating commercially available cellulose acetate membranes with a CO_2_ laser under varied focusing conditions and irradiation passes. Consistent with prior literature studies on bioderived materials, the application of a fire retardant was essential to prevent complete ablation of the CA substrates. The results demonstrate that laser parameters critically influence the nature of the carbon material produced as well as its electrochemical properties. Specifically, double-pass irradiation at a defocus distance of −3 mm and a power of 1.8 W yielded a discretely ordered LIG. In contrast, when double lasing was performed further away from the focal plane at −7.5 mm, activated carbon AC was obtained. This variation is attributed to differences in local temperature profiles during the photothermal pyrolysis process induced by the laser–substrate interaction in conjunction with the P-based fire-retardant treatment. Characterization by SEM, Raman spectroscopy, XPS and TEM confirmed the structural distinctions between LIG and AC. Electrochemical analyses of SCs with electrodes based on these materials revealed that AC electrodes display significant electrical resistance and the analysis on the contribution of width and length resistance was conducted through PEIS measurements. The resistance generally increased with increasing finger length and width, indicating that electrodes with shorter and narrower fingers exhibit superior electrochemical performance. Cyclic voltammetry and galvanostatic charge–discharge assessments confirmed that AC outperforms LIG as an electrode material, likely due to its enhanced capacitive behavior driven by increased structural disorder. The best performing AC device with smaller digits rated more than 63 mF cm^−2^ at 0.1 mA cm^−2^, and an overall energy density of 3.3 µWh cm^−2^ and a maximum power density of 0.42 mW cm^−2^. Overall, this work demonstrates the potential of transforming cellulose acetate, biobased and alternative to fully petroleum-based precursors into LIG. Moreover, it reveals how precise tuning of laser parameters may unlock the synthesis of different carbon-based materials using the same precursor, opening new perspectives for advanced functional material processing methods.

## Author contributions

Angelica Bisceglie: conceptualization, investigation, data curation, formal analysis, visualization, and writing – original draft. Pietro Zaccagnini: conceptualization, formal analysis, methodology, project administration, supervision, validation, and writing – original draft. Luisa Baudino: investigation, data curation, formal analysis, and writing – original draft. Marco Fontana: investigation and writing – review & editing. Yu Kyoung Ryu: conceptualization, project administration, supervision, validation, and writing – review & editing. Andrea Lamberti: funding acquisition, project administration, supervision, validation, and writing – review & editing. Javier Martinez: funding acquisition, project administration, supervision, validation, and writing – review & editing.

## Conflicts of interest

There are no conflicts to declare.

## Supplementary Material

MA-007-D6MA00155F-s001

## Data Availability

The supporting data have been provided as part of the supplementary information (SI). Supplementary information: all supporting data, including design schemes for electrodes and supercapacitor assembly, sample photographs and laser writing parameters, Table S1, additional SEM micrographs, additional Raman spectra and their deconvolutions, Table S2, additional XPS data analysis details, Tables S3 and S4, additional electrochemical analysis and fitting results, Tables S5 and S6. See DOI: https://doi.org/10.1039/d6ma00155f.

## References

[cit1] Nekahi A., Anil Kumar M. R., Deng S., Li X., Petropoulos A., Nanda J., Zaghib K. (2025). Electrochem. Energy Rev..

[cit2] Dissanayake K., Kularatna-Abeywardana D. (2024). J. Energy Storage.

[cit3] Hasan M. M., Haque R., Jahirul M. I., Rasul M. G., Fattah I. M. R., Hassan N. M. S., Mofijur M. (2025). J. Energy Storage.

[cit4] Kumar A., Park E. J., Kim Y. S., Spendelow J. S. (2024). Macromol. Chem. Phys..

[cit5] Wang Y., Zhang L., Hou H., Xu W., Duan G., He S., Liu K., Jiang S. (2021). J. Mater. Sci..

[cit6] Asenbauer J., Eisenmann T., Kuenzel M., Kazzazi A., Chen Z., Bresser D. (2020). Sustainable Energy Fuels.

[cit7] Xu Z. L., Yoon G., Park K. Y., Park H., Tamwattana O., Joo Kim S., Seong W. M., Kang K. (2019). Nat. Commun..

[cit8] Zhai Z., Zhang L., Du T., Ren B., Xu Y., Wang S., Miao J., Liu Z. (2022). Mater. Des..

[cit9] Ponce M. F., Mamani A., Jerez F., Castilla J., Ramos P. B., Acosta G. G., Sardella M. F., Bavio M. A. (2022). Energy.

[cit10] Zhang D., Zhang Y., Liu H., Xu Y., Wu J., Li P. (2023). J. Phys. Chem. Solids.

[cit11] Liu X., Choi J., Xu Z., Grey C. P., Fleischmann S., Forse A. C. (2024). J. Am. Chem. Soc..

[cit12] Liu X., Lyu D., Merlet C., Leesmith M. J. A., Hua X., Xu Z., Grey C. P., Forse A. C. (2024). Science.

[cit13] Guo Y., Liu C., Yin L. X., Zhang X. X., Shan Y. Q., Duan P. G. (2023). J. Anal. Appl. Pyrolysis.

[cit14] Cui C., Qian W., Yu Y., Kong C., Yu B., Xiang L., Wei F. (2014). J. Am. Chem. Soc..

[cit15] Presser V., Heon M., Gogotsi Y. (2011). Adv. Funct. Mater..

[cit16] Gao P. C., Tsai W. Y., Daffos B., Taberna P. L., Pérez C. R., Gogotsi Y., Simon P., Favier F. (2015). Nano Energy.

[cit17] Tong Y., Yang J., Li J., Cong Z., Wei L., Liu M., Zhai S., Wang K., An Q. (2022). J. Mater. Chem. A.

[cit18] Le T. S. D., Phan H. P., Kwon S., Park S., Jung Y., Min J., Chun B. J., Yoon H., Ko S. H., Kim S. W., Kim Y. J. (2022). Adv. Funct. Mater..

[cit19] Jo S. G., Ramkumar R., Lee J. W. (2024). ChemSusChem.

[cit20] Thaweeskulchai T., Sakdaphetsiri K., Schulte A. (2024). Microchim. Acta.

[cit21] Avinash K., Patolsky F. (2023). Mater. Today.

[cit22] Abdulhafez M., Tomaraei G. N., Bedewy M. (2021). ACS Appl. Nano Mater..

[cit23] Kulyk B., Silva B. F. R., Carvalho A. F., Barbosa P., Girão A. V., Deuermeier J., Fernandes A. J. S., Figueiredo F. M. L., Fortunato E., Costa F. M. (2022). Adv. Mater. Technol..

[cit24] Stanford M. G., Yang K., Chyan Y., Kittrell C., Tour J. M. (2019). ACS Nano.

[cit25] Soares R. R. A., Hjort R. G., Pola C. C., Parate K., Reis E. L., Soares N. F. F., Mclamore E. S., Claussen J. C., Gomes C. L. (2020). ACS Sens..

[cit26] Yi J., Chen J., Yang Z., Dai Y., Li W., Cui J., Ciucci F., Lu Z., Yang C. (2019). Adv. Energy Mater..

[cit27] Reina M., Serrapede M., Zaccagnini P., Pedico A., Castellino M., Bianco S., Ouisse T., Pazniak H., Gonzalez-Julian J., Lamberti A. (2023). Electrochim. Acta.

[cit28] Zaccagnini P., Tien Y., Baudino L., Pedico A., Bianco S., Lamberti A. (2023). Adv. Mater. Technol..

[cit29] Chen B., Johnson Z. T., Sanborn D., Hjort R. G., Garland N. T., Soares R. R. A., Van Belle B., Jared N., Li J., Jing D., Smith E. A., Gomes C. L., Claussen J. C. (2022). ACS Nano.

[cit30] Stanford M. G., Li J. T., Chyan Y., Wang Z., Wang W., Tour J. M. (2019). ACS Nano.

[cit31] Lin J., Peng Z., Liu Y., Ruiz-Zepeda F., Ye R., Samuel E. L. G., Yacaman M. J., Yakobson B. I., Tour J. M. (2014). Nat. Commun..

[cit32] Beckham J. L., Li J. T., Stanford M. G., Chen W., McHugh E. A., Advincula P. A., Wyss K. M., Chyan Y., Boldman W. L., Rack P. D., Tour J. M. (2021). ACS Nano.

[cit33] Lamberti A., Serrapede M., Ferraro G., Fontana M., Perrucci F., Bianco S., Chiolerio A., Bocchini S. (2017). 2D Mater..

[cit34] Bressi A. C., Dallinger A., Steksova Y., Greco F. (2023). ACS Appl. Mater. Interfaces.

[cit35] Lengger S. K., Neumaier L., Haiden L., Feuchter M., Griesser T., Kosel J. (2024). Sustainable Mater. Technol..

[cit36] Nam H. K., Le T. S. D., Yang D., Kim B., Lee Y., Hwang J. S., Kim Y. R., Yoon H., Kim S. W., Kim Y. J. (2023). Adv. Mater. Technol..

[cit37] Chyan Y., Ye R., Li Y., Pratap Singh S., Arnusch C. J., Tour J. M. (2018). ACS Nano.

[cit38] Imbrogno A., Islam J., Santillo C., Castaldo R., Sygellou L., Larrigy C., Murray R., Vaughan E., Hoque M. K., Quinn A. J., Iacopino D. (2022). ACS Appl. Electron. Mater..

[cit39] Chyan Y., Cohen J., Wang W., Zhang C., Tour J. M. (2019). ACS Appl. Nano Mater..

[cit40] Vaughan E., Santillo C., Imbrogno A., Gentile G., Quinn A. J., Kaciulis S., Lavorgna M., Iacopino D. (2023). ACS Sustainable Chem. Eng..

[cit41] Kulyk B., Silva B. F. R., Carvalho A. F., Silvestre S., Fernandes A. J. S., Martins R., Fortunato E., Costa F. M. (2021). ACS Appl. Mater. Interfaces.

[cit42] Pinheiro T., Silvestre S., Coelho J., Marques A. C., Martins R., Sales M. G. F., Fortunato E. (2021). Adv. Mater. Interfaces.

[cit43] Hamidi H., Levieux J., Larrigy C., Russo A., Vaughan E., Murray R., Quinn A. J., Iacopino D. (2023). Biosens. Bioelectron. X.

[cit44] Araújo M. C. B., Costa M. F. (2019). Environ. Res..

[cit45] Yousefi Nasab A., Oskoei V., Rezanasab M., Alinejad N., Hosseinzadeh A., Kashi G. (2022). Environ. Sci. Pollut. Res..

[cit46] Torkashvand J., Saeedi-Jurkuyeh A., Rezaei Kalantary R., Gholami M., Esrafili A., Yousefi M., Farzadkia M. (2022). Sci. Rep..

[cit47] De Fenzo A., Giordano M., Sansone L. (2020). Materials.

[cit48] Dubrovina L., Naboka O., Ogenko V., Gatenholm P., Enoksson P. (2014). J. Mater. Sci..

[cit49] Fischer J., Thümmler K., Fischer S., Gonzalez Martinez I. G., Oswald S., Mikhailova D. (2021). Energy Fuels.

[cit50] Wojdyr M. (2010). J. Appl. Crystallogr..

[cit51] Claramunt S., Varea A., López-Díaz D., Velázquez M. M., Cornet A., Cirera A. (2015). J. Phys. Chem. C.

[cit52] López-Díaz D., López Holgado M., García-Fierro J. L., Velázquez M. M. (2017). J. Phys. Chem. C.

[cit53] Brubaker Z. E., Miskowiec A., Niedziela J. L. (2022). Phys. Rev. Mater..

[cit54] Brubaker Z. E., Langford J. J., Kapsimalis R. J., Niedziela J. L. (2021). J. Mater. Sci..

[cit55] Sadezky A., Muckenhuber H., Grothe H., Niessner R., Pöschl U. (2005). Carbon.

[cit56] Fairley N., Fernandez V., Richard-Plouet M., Guillot-Deudon C., Walton J., Smith E., Flahaut D., Greiner M., Biesinger M., Tougaard S., Morgan D., Baltrusaitis J. (2021). Appl. Surf. Sci. Adv..

[cit57] Ferrari A. C., Basko D. M. (2013). Nat. Nanotechnol..

[cit58] Ferrari A. C., Robertson J. (2001). Phys. Rev. B: Condens. Matter Mater. Phys..

[cit59] Croce A., Re G., Bisio C., Gatti G., Coluccia S., Marchese L. (2021). Res. Chem. Intermed..

[cit60] Bin Wu J., Lin M. L., Cong X., Liu H. N., Tan P. H. (2018). Chem. Soc. Rev..

[cit61] Shimodaira N., Masui A. (2002). J. Appl. Phys..

[cit62] Vali I. P., Anusha B. S., Pruthvija M., Savitha S., Ravindra S., Nagaveni M., Poojitha P. S., Swathi N. (2024). Mater. Chem. Phys..

[cit63] Mahmood F., Mahmood F., Zhang H., Lin J., Wan C. (2020). ACS Omega.

[cit64] Kulyk B., Matos M., Silva B. F. R., Carvalho A. F., Fernandes A. J. S., Evtuguin D. V., Fortunato E., Costa F. M. (2022). Diamond Relat. Mater..

[cit65] Lamberti A., Perrucci F., Caprioli M., Serrapede M., Fontana M., Bianco S., Ferrero S., Tresso E. (2017). Nanotechnology.

[cit66] Gupta R., Gupta A., George J. K., Verma N. (2025). Colloids Surf., A.

[cit67] Cançado L. G., Jorio A., Ferreira E. H. M., Stavale F., Achete C. A., Capaz R. B., Moutinho M. V. O., Lombardo A., Kulmala T. S., Ferrari A. C. (2011). Nano Lett..

[cit68] Ni Z. H., Yu T., Lu Y. H., Wang Y. Y., Feng Y. P., Shen Z. X. (2008). ACS Nano.

[cit69] Larrigy C., Burke M., Imbrogno A., Vaughan E., Santillo C., Lavorgna M., Sygellou L., Paterakis G., Galiotis C., Iacopino D., Quinn A. J. (2023). Adv. Mater. Technol..

[cit70] Biesinger M. C. (2022). Appl. Surf. Sci..

[cit71] Bezinge L., Lesinski J. M., Suea-Ngam A., Richards D. A., de Mello A. J., Shih C. J. (2023). Adv. Mater..

[cit72] Mahmood F., Zhang C., Xie Y., Stalla D., Lin J., Wan C. (2019). RSC Adv..

[cit73] Sankaran S. T., Dallinger A., Bressi A. C., Marino A., Ciofani G., Szkudlarek A., Bilovol V., Sokolowski K., Kunert B., Hampel H. K., Bernal H. G., Greco F. (2024). Small.

[cit74] Serbanescu O. S., Pandele A. M., Miculescu F., Voicu S. I. (2020). Coatings.

[cit75] Movaghgharnezhad S., Kim M., Min Lee S., Jeong H., Kim H., Gak Kim B., Kang P. (2023). Mater. Des..

[cit76] Dsoke S., Tian X., Täubert C., Schlüter S., Wohlfahrt-Mehrens M. (2013). J. Power Sources.

[cit77] Lazanas A. C., Prodromidis M. I. (2023). ACS Meas. Sci. Au.

[cit78] Liu H., Song H., Chen X., Zhang S., Zhou J., Ma Z. (2015). J. Power Sources.

[cit79] Silvestre S. L., Pinheiro T., Marques A. C., Deuermeier J., Coelho J., Martins R., Pereira L., Fortunato E. (2022). Flexible Printed Electron..

[cit80] Coelho J., Correia R. F., Silvestre S., Pinheiro T., Marques A. C., Correia M. R. P., Pinto J. V., Fortunato E., Martins R. (2023). Microchim. Acta.

[cit81] Le T. S. D., Lee Y. A., Nam H. K., Jang K. Y., Yang D., Kim B., Yim K., Kim S. W., Yoon H., Kim Y. J. (2022). Adv. Funct. Mater..

[cit82] Reina M., Scalia A., Auxilia G., Fontana M., Bella F., Ferrero S., Lamberti A. (2022). Adv. Sustainable Syst..

[cit83] Shen C., Xu S., Xie Y., Sanghadasa M., Wang X., Lin L. (2017). J. Microelectromech. Syst..

[cit84] Sangeetha D. N., Selvakumar M. (2018). Appl. Surf. Sci..

[cit85] Nwanya A. C., Musheghyan-Avetisyan A., György E., Pérez del Pino Á. (2024). Surf. Interfaces.

