# Supplementary material for: Laser-induced carbonization of cellulose acetate for controlled carbon structure evolution
Source: Mater Adv. 2026 Aug 3. Online ahead of print. doi: 10.1039/d6ma00155f (PMC13430549; doi:10.1039/d6ma00155f)
Supplement: MA-OLF-D6MA00155F-s001 [file MA-OLF-D6MA00155F-s001.pdf]

Supplementary Information (SI) for Materials Advances.
This journal is © The Royal Society of Chemistry 2026

SUPPORTING INFORMATION

Laser-Induced Carbonization of Cellulose Acetate for Controlled Carbon Structure Evolution

Angelica Bisceglie[ab], Pietro Zaccagnini[ab], Luisa Baudino[ab], Marco Fontana[ab], Yu Kyoung Ryu[cd]*, Andrea Lamberti[ab]*, Javier Martinez[ce]

[a]Department of Applied Science and Technologies – Polytechnic of Turin, Corso Duca degli Abruzzi 24, Turin 10129, Italy

[b]Centre for Sustainable and Future Technologies – Italian Institute of Technology, Via Livorno 60, Turin 10144, Italy

[c]Instituto de Sistemas Optoelectrónicos y Microtecnología, Universidad Politécnica de Madrid, Avenida Complutense 30, Madrid 28040, Spain

[d]Departamento de Física Aplicada e Ingeniería de Materiales, E.T.S.I Industriales, Universidad Politécnica de Madrid, C/ José Gutiérrez Abascal 2, Madrid 28006, Spain

[e]Departamento de Ciencia de Materiales-CIME, E.T.S.I Caminos, Canales y Puertos, Universidad Politécnica de Madrid, C/ Profesor Aranguren s/n, 28040 Madrid, Spain

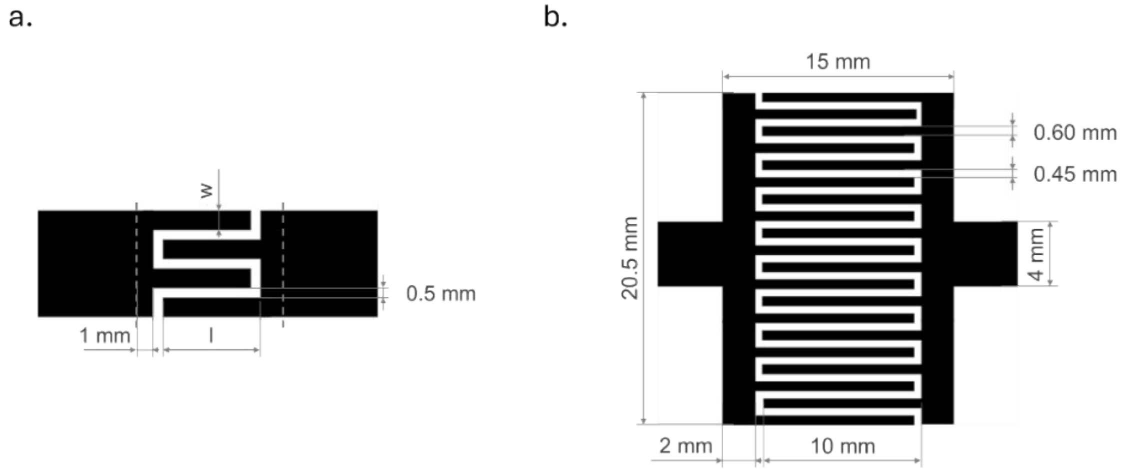

Fig. S1. Interdigitated electrodes design scheme used for (a) P2-3 lasing conditions and (b) P2-7.5 parameters. In (b) l is the fingers' length, varied between 1 and 5 in 2 mm increments; w is the fingers' width, which was 0.5 mm or 1 mm. The area between the dashed lines (fingers + 1 mm) is the one exposed to the electrolyte.

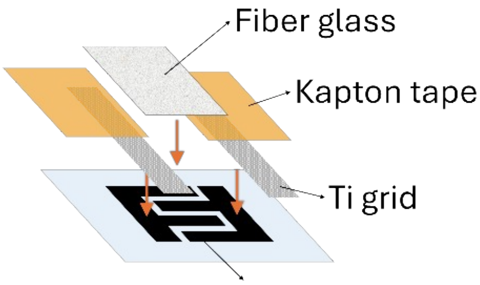

Fig. S2. Scheme of supercapacitors assembly. Titanium grids served as current collectors for both electrodes. Kapton tape was applied to prevent direct contact between the current collectors and the electrolyte. Additionally, fiberglass was used to hold the electrolyte in place and minimize its evaporation.

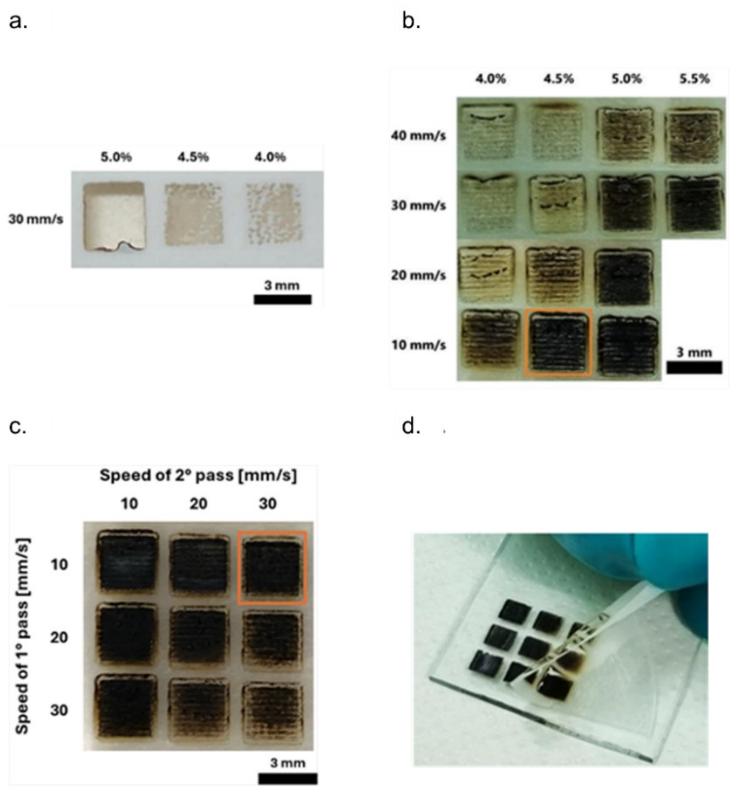

Fig. S3. Photographs of samples obtained after in-focus irradiation of (a) untreated CA membranes at different lasing powers. (b) Treated membranes exposed to single lasing pass at different lasing power and speed conditions. (c) Treated membranes exposed to double lasing pass using the same lasing power and different speed conditions for the first and the second irradiation step. Samples highlighted by orange square are the ones that yield the optimal results. (d) Photograph showing the easy removal of the untransformed cellulose acetate membrane from the laser-treated areas.

Table S1. Laser operating parameters. Nominal laser power, scanning speed, and distance from the focal plane are here reported. Corresponding fluence values were derived using a geometrical approximation of the beam expansion at varying distances.

Sample name	Number of passes	Distance from focal plane [mm]	Power [W]	Speed [mm/s]	Fluence [J/cm^2]
P1-7.5	1	7.5	1.8	10	118
P1-4	1	4			140
P1-3	1	3			149
P1-0	1	0			180
P2-7.5	1st	7.5		10	157
	2nd			30	
P2-4	1st	4		10	187
	2nd			30	
P2-3	1st	3		10	198
	2nd			30	
P1-0	1st	0		10	240
	2nd			30	

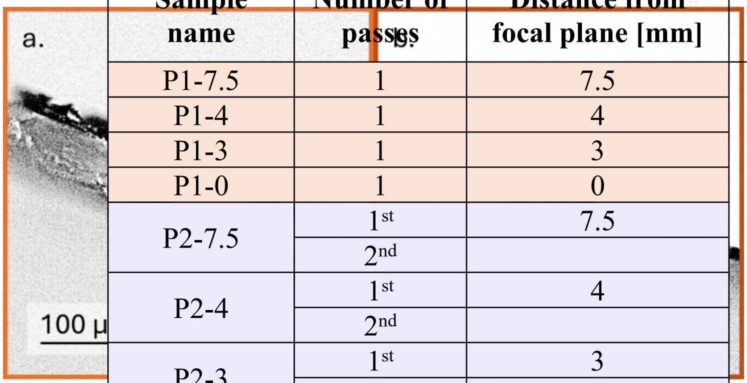

Fig. S4. Optical microscopy cross-section images of sample (a) P2-3, (b) P2-7.5. The average thickness is 23.85 ± 5.35 μm for sample P2-3 and 16.02 ± 2.00 μm for sample P2-7.5 (average values calculated over 10 measurements).

At greater distances from the focal plane (P1-7.5), the material experiences reduced fluence levels, resulting in a morphology that is largely similar to that of the untreated membrane (see main text, **Fig. 1**). As the lasing plane approaches the focal plane, the fluence increases, facilitating a more pronounced transformation of the material. All samples fabricated closer to the focal plane (P1-4, P1-3, P1-focused) exhibit high particle aggregation, with continuous surfaces characterized by few open pores and some protrusions likely resulting from closed porosity, which may be retraced to GO formation. During the second laser pass, additional energy is delivered onto the carbonaceous structures previously formed. As discussed in the main text, the sample prepared farther from the focal plane (P2-7.5) is activated carbon, exhibiting a rough surface in the high-magnification SEM micrographs. Samples fabricated at or near the focal plane appear more porous, a consequence of the intensive degassing processes resulting from the formation of LIG upon GO reduction. The P2-focused sample, produced exactly at the focal plane, appears denser and exhibits less pronounced porosity than the P2-4 and P2-3 samples, which were fabricated under defocused conditions. At lower magnification, these latter samples display remarkable similarities, notably highly porous architectures comprised of thin, interconnected network-like features. Under higher magnification, both demonstrate increased surface roughness compared to sample P2-7.5, which is distinguished by its relatively smooth surface.

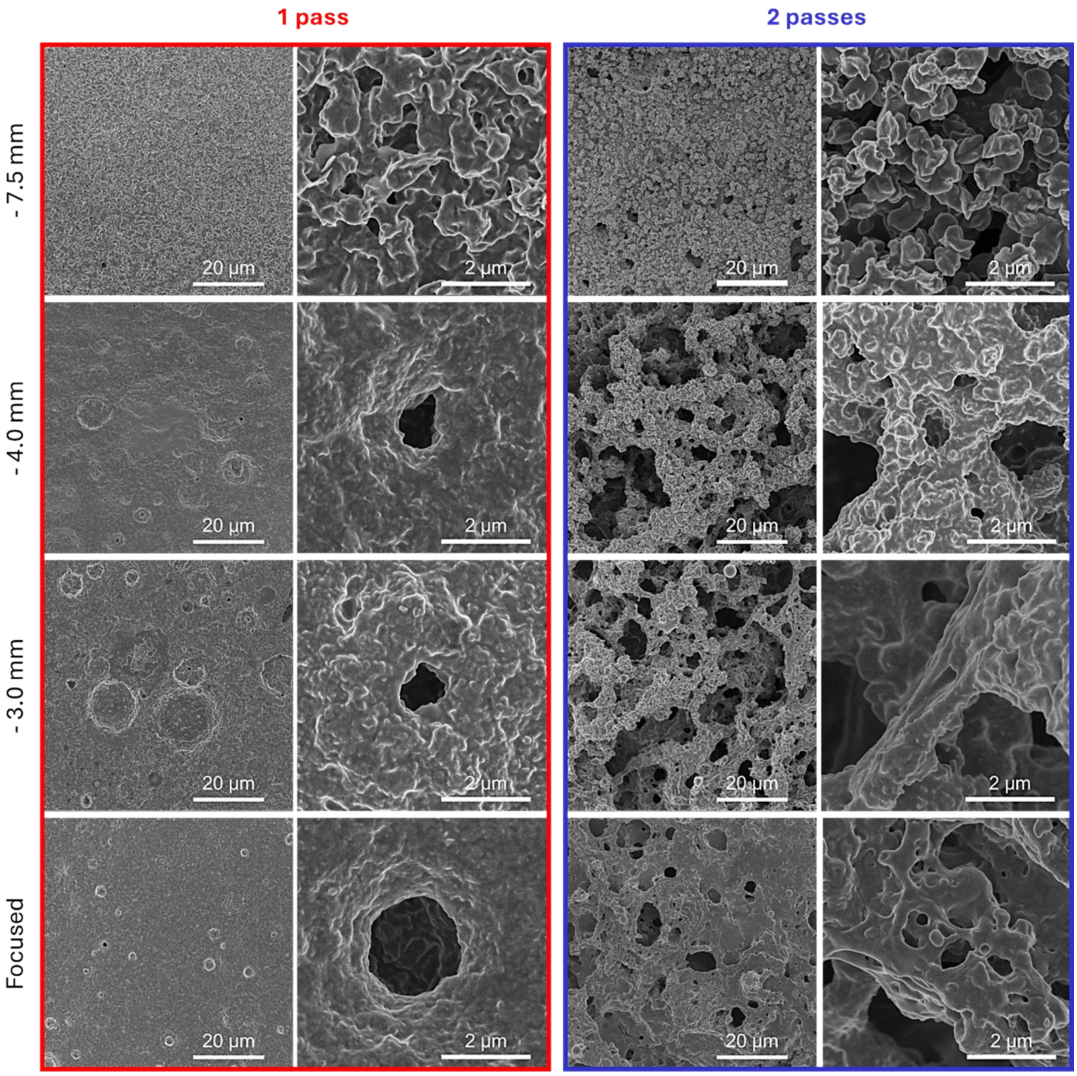

Fig. S5. SEM micrographs at magnifications of 1200x and 15000x of samples obtained at different distances from the focal plane, employing single and double lasing steps.

Raman spectral analysis of samples subjected to a single lasing pass reveals the absence of the characteristic 2D peak associated with graphene-like structures. Notably, the spectrum of sample P1-7.5 is indicative of amorphous carbon formation, as discussed in the main text. Conversely, samples P1-4, P1-3, and P1-focused exhibit distinct and well-defined D and G peaks, suggesting the formation of graphene oxide (GO). It should be emphasized that the interpretations presented for P1-4 in the main text are equally applicable to P1-3 and P1-focused. Following a second laser pass, the Raman spectrum of sample P2-7.5 displays overlapping D and G peaks along with a broad and poorly resolved 2D band, indicative of enhanced structural ordering compared to the initially formed amorphous carbon. However, the structure remains predominantly disordered, as detailed further in the main text. Similarly, the spectral features observed for P2-4 are consistent with those recorded for P2-3 and P2-focused. In particular, all samples fabricated near the focal plane exhibit sharp, well-resolved D and G peaks with minimal overlap, alongside the presence of a D' peak, collectively indicating an increased degree of structural order. Moreover, the clear emergence of a well-defined 2D peak supports the hypothesis of the formation of LIG.

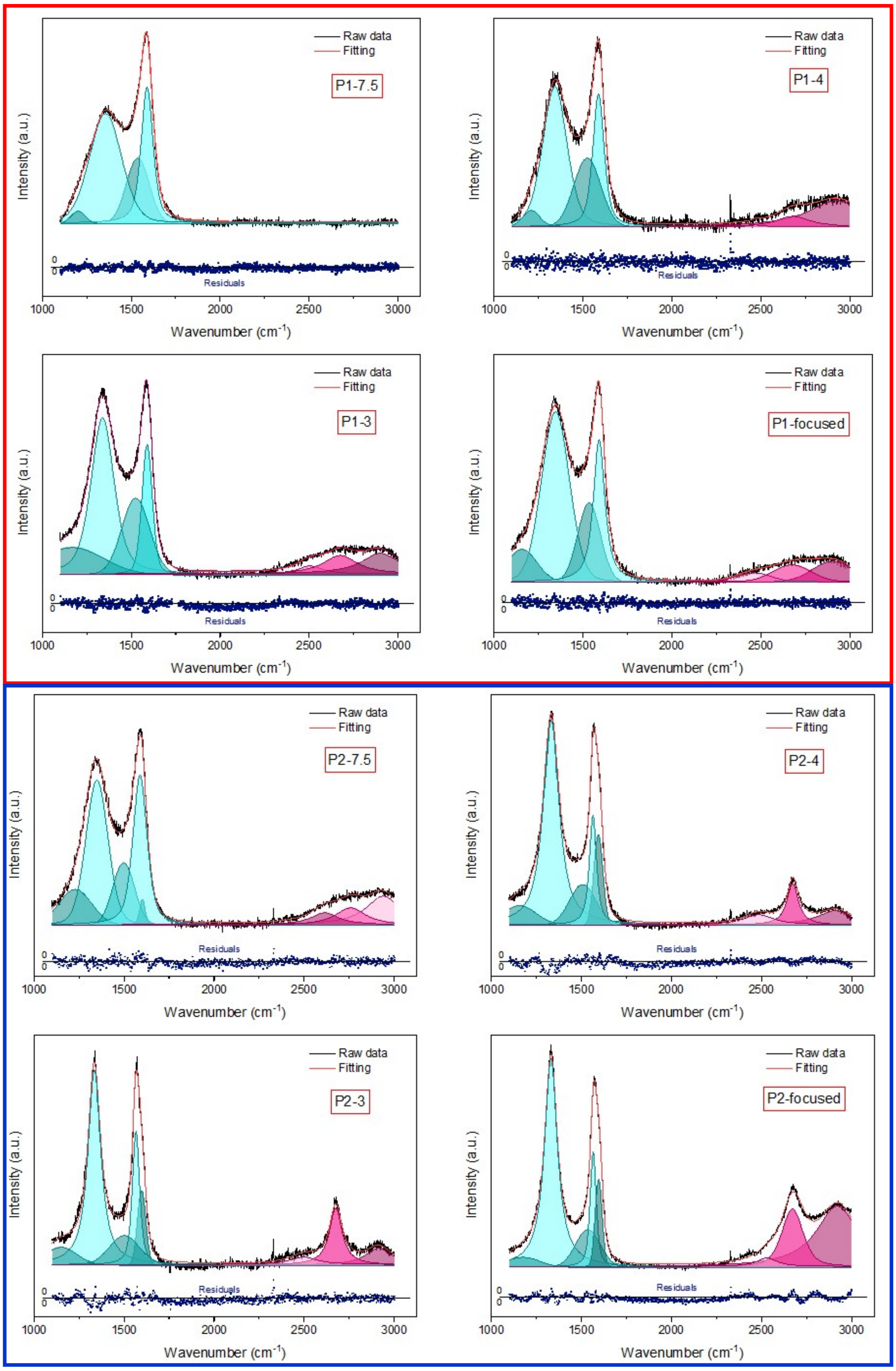

Fig. S6. Deconvoluted Raman spectra of samples produced with single and double lasing pass. Peaks used for their deconvolution are represented in green tones for the first order region, pink tones for the second order region. The red line represents the function that best fits the Raman signal. Residuals, calculated by subtracting the fitted curve from the raw data, are shown at the bottom.

Table S2. I_D/I_G ratios calculated for samples produced in single and double irradiation steps.

Sample	I_D/I_G
P1-7.5	0.81
P1-4	1.21
P1-3	1.06
P1-focused	1.20
P2-7.5	0.97
P2-4	1.46
P2-3	1.34
P2-focused	1.81

Further analysis of the Raman spectra confirms that this second lasing pass facilitates significant ordering within the material's structure. The halving of the FWHM values for the D and G peaks after the second lasing pass in samples produced closer to the focal plane (P2-4, P2-3, P2-focused) indicates an increase in the size of *sp²* clusters, suggesting that the additional pass induces greater structural order through thermal effects on the material's surface. In contrast, for samples P1-7.5 and P2-7.5, the FWHM remains nearly unchanged, indicating little to no variation in *sp²* cluster size between the single- and double-pass methods.

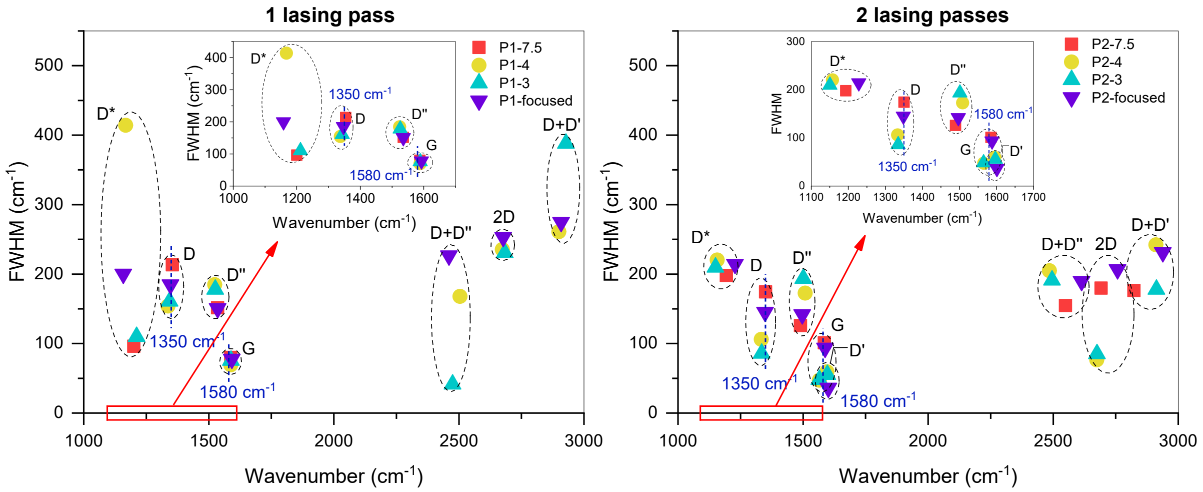

Fig. S7. Correlation between peak position and FWHM. Dashed lines represent the theoretical positions of D and G peaks in graphitic materials for samples produced both in single and double pass. The inset provides an enlarged view of the first order region.

Table S3. Samples surface composition from XPS survey analyses.

	C (at.%)	O (at.%)	P (at.%)	N (at.%)	Si (at.%)
P1-3	56.6 ± 0.5	37.6 ± 0.4	4.2 ± 0.2	1.0 ± 0.3	0.6 ± 0.1
P1-7.5	55.6 ± 0.4	39.0 ± 0.4	3.72 ± 0.1	1.1 ± 0.2	0.6 ± 0.1
P2-3	33.4 ± 0.9	50.6 ± 0.8	10.4 ± 0.4	3.1 ± 0.5	2.5 ± 0.3
P2-7.5	25.7 ± 0.5	56.5 ± 0.5	11.50 ± 0.2	4.1 ± 0.3	2.2 ± 0.2

Table S4. Detailed deconvolution of the normalized HR C 1s peaks. The asymmetric C–C *sp*^{2} peak in graphitic structures (284.5 eV) was used as reference for the calibration. GL(30) line shapes were used for all components, except for the asymmetric graphitic carbon for which LA(1.2,2.5,5) was used. [1]

		C *sp*^{2}	C *sp*^{3}	C-O	C=O	COOH	Sat.
CA	*peak position*	284.5 eV	285.0 eV	286.5 eV	287.5 eV	289.0 eV	291.3 eV
	%	-	24.07%	45.19%	5.01%	25.73%	-
P1-3	*peak position*	284.5 eV	285.0 eV	286.6 eV	287.5 eV	289.1 eV	291.3 eV
	%	6.5%	58.6%	14.9%	3.3%	14.5%	2.2%
P2-3	*peak position*	284.5 eV	285.0 eV	286.5 eV	287.7 eV	289.1 eV	291.3 eV
	%	13.8%	54.2 %	10.5%	4.8%	11.7%	5.0%
P2-7.5	*peak position*	284.5 eV	285.0 eV	286.6 eV	287.6 eV	289.1 eV	291.3 eV
	%	5.7%	57.8%	17.1%	5.4%	12.4%	1.6%

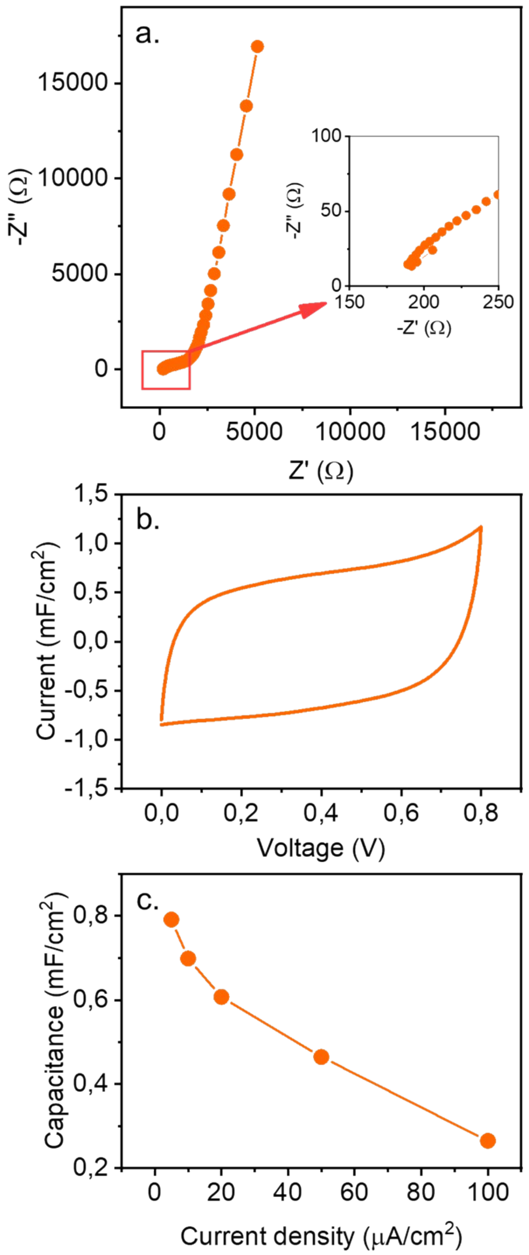

Fig. S8. Electrochemical characterization of devices using P2-3 as electrode material. (a) Nyquist plot, (b) CV curve recorded at 10 mV s^{-1}, (c) specific capacitances from GCD curves.

EIS measurements were fitted by means of an equivalent circuit proposed in a previous work [2] and reported in the following scheme. The circuit represents an extension of the model proposed by Dsoke et al.[3], in which the series of the Warburg element exploited to describe diffusion limitations, and the CPE exploited to describe the cell non ideal capacitance are substituted by the anomalous diffusion element by Bisquert.[4] According to our experimental data, the electrodes parasitic behavior could be accounted for by two different R//CPE elements owing to distinguishable processes, contrary to the model proposed by Dsoke. These occur in the medium to high frequencies and can be attributed to the active material non uniform phase as well as contact resistance due to the Ti grid. The Bisquert element is related to anomalous diffusion accounts for the ions' diffusion limitations within porous structures and finally for the EDL formation, or generally adsorption processes, thanks to its reflective boundary condition. Because of this reason, as proposed in our previous work, the cell capacitance can be calculated out of the Bisquert element considering the

characteristic time, t_5, as the time constant for the device charging. The results are in accordance with the specific areal capacitance trends derived from CV and GCD experiments.

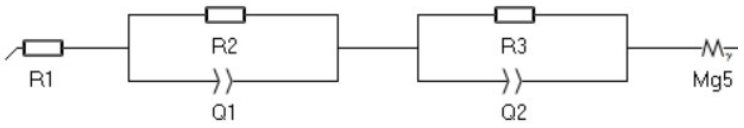

Fig. S9. Circuit used to fit Nyquist plots.

The fitting results are reported in the following **Table S4**. According to our analyses, overall, the series resistance increases in both cases of narrow and large digit dimensions with increasing digit lengths, proving that ESR is mainly dominated by the carbon phase and follows Ohm's law predictions. Electrodes capacitances decrease with both digit lengths and widths implying that probably some portions of the digits behave as dead surface not contributing to the overall device capacitance. To give further reading to these results, we decided to average the fitting values in order to study possible trends per digit length. The results of this operation are reported in **Table S5**. The results of these analyses confirm that the series resistance is dominated by the digit length while device nominal capacitances decrease linearly with digit length. These results are in line with the trends reported in the GCD analyses. The main differences in areal capacitance with respect to GCD data are ascribable to the pseudocapacitive contributions that are not measured at device open circuit voltage. These contributions to the overall device capacitance derive from the surface chemistry of the analyzed carbon-based devices. Interestingly, the areal capacitance decreases logarithmically as a function of the digit length. At the moment, the authors are not able to provide explanations for this trend. These results suggest that in principle, the interdigitated device design based on resistive samples must follow the rule of short digit length, especially in the case of resistive materials electrode not supported by metallic current collectors. These conclusions can be further claimed by observing the results related to the anomalous diffusion element. Despite the diffusion resistance values, probably due to combined solid and liquid state conductivity, the overall particles diffusion characteristic time is such that the resulting capacitance follows the inverse trend of digit lengths. Another interesting correlation was noted by observing the anomalous diffusion parameter. According to the ADIb in [3], by observing the gamma values these are not correlated to the device overall resistance but rather to the digits' length and the possible explanation could be that at reduced digit length, the increased dose due to more frequent passes could have induced more disordered phase causing sub diffusive effect and further increased capacitance.

Table S5. EIS fitting results obtained by exploiting the equivalent circuit proposed in Figure S8

**L**	**W**	**Rs**	**Rp1**	**Q1**		**Rp2**	**Q2**		**Mg**			**C**	**S**	**C_A**
mm	mm	Ω	Ω	F s^(a^{-1})	a1	Ω	F s^(a-1)	a1	Ω	s	γ	mF	cm^2	mF cm^{-2}
1	0.5	65.4	177.2	1.98E-05	0.59	278.8	2.46E-04	0.53	12197	65.21	0.56	5.3	0.09	59,4
1	1	81.7	146.6	7.76E-06	0.66	122.0	7.20E-05	0.65	7514	54.66	0.74	7.3	0.15	48,5
3	0.5	104.1	260.7	3.65E-04	0.55	129.3	1.10E-05	0.62	3184	12.62	0.66	4.0	0,13	30,5
3	1	94.3	180.3	3.76E-05	0.51	173.5	1.99E-04	0.70	2347	16.63	0.70	7.1	0,23	30,8
5	0.5	141.1	332.0	1.59E-05	0.90	384.0	2.97E-05	0.50	6475	20.60	0.83	3.2	0,17	18,7
5	1	111.7	82.9	5.54E-04	0.58	174.6	1.06E-05	0.62	3175	20.96	0.76	6.6	0,31	21,3
LIG		161.4	1399.7	9.01E-05	0.41	2688.4	4.04E-03	1	3581	3.84	0.76	1.0	2,02	0,5

Table S6. EIS Fitting results parameters averaged over the digit length and different width

L	W	Rs	Rp1	Q1		Rp2	Q2		Mg			C	C_A
mm	mm	Ω	Ω	F s^(a^{-1})	a1	Ω	F s^(a^{-1})	a1	Ω	s	γ	mF	mF cm^{-2}
1	0.5	73.5	146.6	1.38E-05	0.62	200.4	1.59E-04	0.59	9856	59.94	0.65	6.3	54.0
	1												
3	0.5	99.2	260.7	2.01E-04	0.53	151.4	1.05E-04	0.66	2766	14.63	0.68	5.5	30.6
	1												
5	0.5	126.4	332.0	2.85E-04	0.74	279.3	2.02E-05	0.56	4825	20.78	0.80	4.9	20.0
	1												

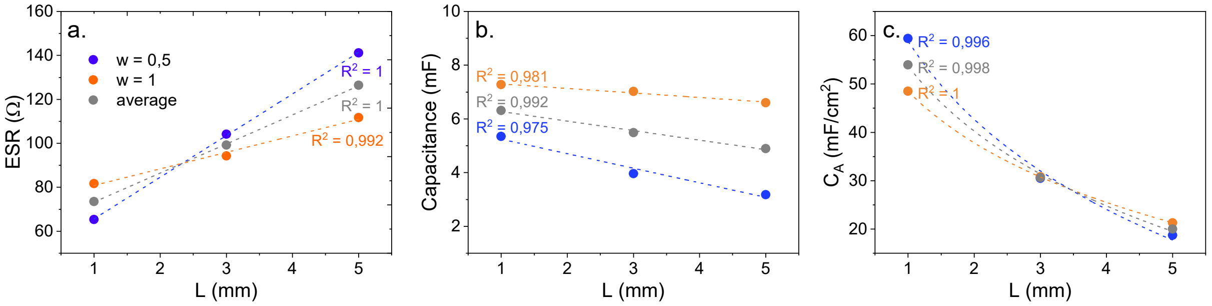

Fig. S10. Correlation of (a) ESRs (b) capacitances and (c) specific capacitances derived from Nyquist plots and reported in *Table*. The first two display a linear trend with fingers' length, the last one a logarithmic trend. Devices capacitances were derived as t_3/R_{d3}.

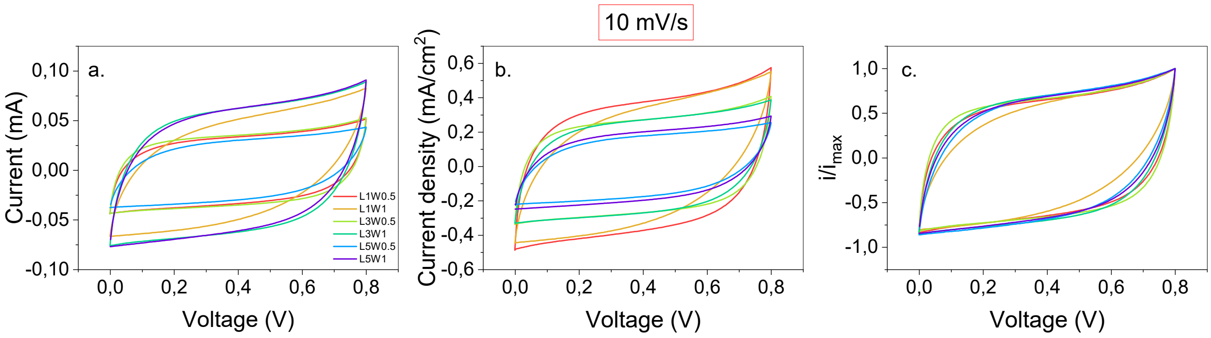

Fig. S11. CV curves represented in different ways. (a) CV curves as measured experimentally; (b) CV curves plotted as current density, with current normalized to the electrode area exposed to the electrolyte; (c) CV curves with current normalized to the maximum measured value; (d) CV curves with current divided by the scan rate, providing indication of capacitance.

References

1. Biesinger, M. C. (2022). Accessing the robustness of adventitious carbon for charge referencing (correction) purposes in XPS analysis: Insights from a multi-user facility data review. *Applied Surface Science*, *597*, 153681. https://doi.org/10.1016/J.APSUSC.2022.153681

2. Zaccagnini, P.; Tien, Y.; Baudino, L.; Pedico, A.; Bianco, S.; Lamberti, A. Optimization of Laser-Induced Graphene Electrodes for High Voltage and Highly Stable Microsupercapacitors. *Adv Mater Technol* **2023**, 8 (23). https://doi.org/10.1002/admt.202300833.

3. Dsoke, S.; Tian, X.; Täubert, C.; Schlüter, S.; Wohlfahrt-Mehrens, M. Strategies to Reduce the Resistance Sources on Electrochemical Double Layer Capacitor Electrodes. *J Power Sources* **2013**, 238, 422–429. https://doi.org/10.1016/j.jpowsour.2013.04.031.

4. Bisquert, J.; Compte, A. Theory of the Electrochemical Impedance of Anomalous Diffusion. *Journal of Electroanalytical Chemistry* **2001**, 499, 112–120. https://doi.org/10.1016/S0022-0728(00)00497-6
